# Tackling Atherosclerosis via Selected Nutrition

**DOI:** 10.3390/ijms23158233

**Published:** 2022-07-26

**Authors:** Anna Vesnina, Alexander Prosekov, Victor Atuchin, Varvara Minina, Anastasia Ponasenko

**Affiliations:** 1 Laboratory of Natural Nutraceuticals Biotesting, Research Department, Kemerovo State University, 650043 Kemerovo, Russia; koledockop1@mail.ru; 2 Laboratory of Biocatalysis, Kemerovo State University, 650043 Kemerovo, Russia; aprosekov@rambler.ru; 3 Laboratory of Optical Materials and Structures, Institute of Semiconductor Physics, 630090 Novosibirsk, Russia; 4 Research and Development Department, Kemerovo State University, 650000 Kemerovo, Russia; 5 Laboratory of Applied Physics, Novosibirsk State University, 630090 Novosibirsk, Russia; 6 Department of Industrial Machinery Design, Novosibirsk State Technical University, 630073 Novosibirsk, Russia; 7 R&D Center “Advanced Electronic Technologies”, Tomsk State University, 634034 Tomsk, Russia; 8 Department of Genetic and Fundamental Medicine, Kemerovo State University, 650000 Kemerovo, Russia; vminina@mail.ru; 9 Laboratory of Genome Medicine, Research Institute for Complex Issues of Cardiovascular Diseases, 650002 Kemerovo, Russia; avapanass@mail.ru

**Keywords:** atherosclerosis, personalized nutrition, nutritional genetics, nutrient, gastrointestinal microbiota, epigenetics, chrono-nutrition, nutrigenetics, nutrigenomics, animal models of human atherosclerosis

## Abstract

The development and pathogenesis of atherosclerosis are significantly influenced by lifestyle, particularly nutrition. The modern level of science and technology development promote personalized nutrition as an efficient preventive measure against atherosclerosis. In this survey, the factors were revealed that contribute to the formation of an individual approach to nutrition: genetic characteristics, the state of the microbiota of the gastrointestinal tract (GIT) and environmental factors (diets, bioactive components, cardioprotectors, etc.). In the course of the work, it was found that in order to analyze the predisposition to atherosclerosis associated with nutrition, genetic features affecting the metabolism of nutrients are significant. The genetic features include the presence of single nucleotide polymorphisms (SNP) of genes and epigenetic factors. The influence of telomere length on the pathogenesis of atherosclerosis and circadian rhythms was also considered. Relatively new is the study of the relationship between chrono-nutrition and the development of metabolic diseases. That is, to obtain the relationship between nutrition and atherosclerosis, a large number of genetic markers should be considered. In this relation, the question arises: “How many genetic features need to be analyzed in order to form a personalized diet for the consumer?” Basically, companies engaged in nutrigenetic research and choosing a diet for the prevention of a number of metabolic diseases use SNP analysis of genes that accounts for lipid metabolism, vitamins, the body’s antioxidant defense system, taste characteristics, etc. There is no set number of genetic markers. The main diets effective against the development of atherosclerosis were considered, and the most popular were the ketogenic, Mediterranean, and DASH-diets. The advantage of these diets is the content of foods with a low amount of carbohydrates, a high amount of vegetables, fruits and berries, as well as foods rich in antioxidants. However, due to the restrictions associated with climatic, geographical, material features, these diets are not available for a number of consumers. The way out is the use of functional products, dietary supplements. In this approach, the promising biologically active substances (BAS) that exhibit anti-atherosclerotic potential are: baicalin, resveratrol, curcumin, quercetin and other plant metabolites. Among the substances, those of animal origin are popular: squalene, coenzyme Q10, omega-3. For the prevention of atherosclerosis through personalized nutrition, it is necessary to analyze the genetic characteristics (SNP) associated with the metabolism of nutrients, to assess the state of the microbiota of the GIT. Based on the data obtained and food preferences, as well as the individual capabilities of the consumer, the optimal diet can be selected. It is topical to exclude nutrients of which their excess consumption stimulates the occurrence and pathogenesis of atherosclerosis and to enrich the diet with functional foods (FF), BAS containing the necessary anti-atherosclerotic, and stimulating microbiota of the GIT nutrients. Personalized nutrition is a topical preventive measure and there are a number of problems hindering the active use of this approach among consumers. The key factors include weak evidence of the influence of a number of genetic features, the high cost of the approach, and difficulties in the interpretation of the results. Eliminating these deficiencies will contribute to the maintenance of a healthy state of the population through nutrition.

## 1. Introduction

**Figure 1:**
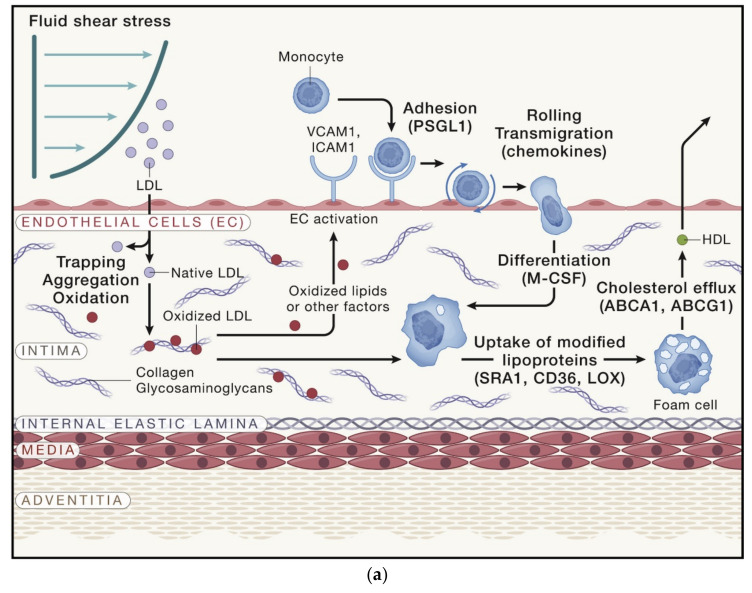

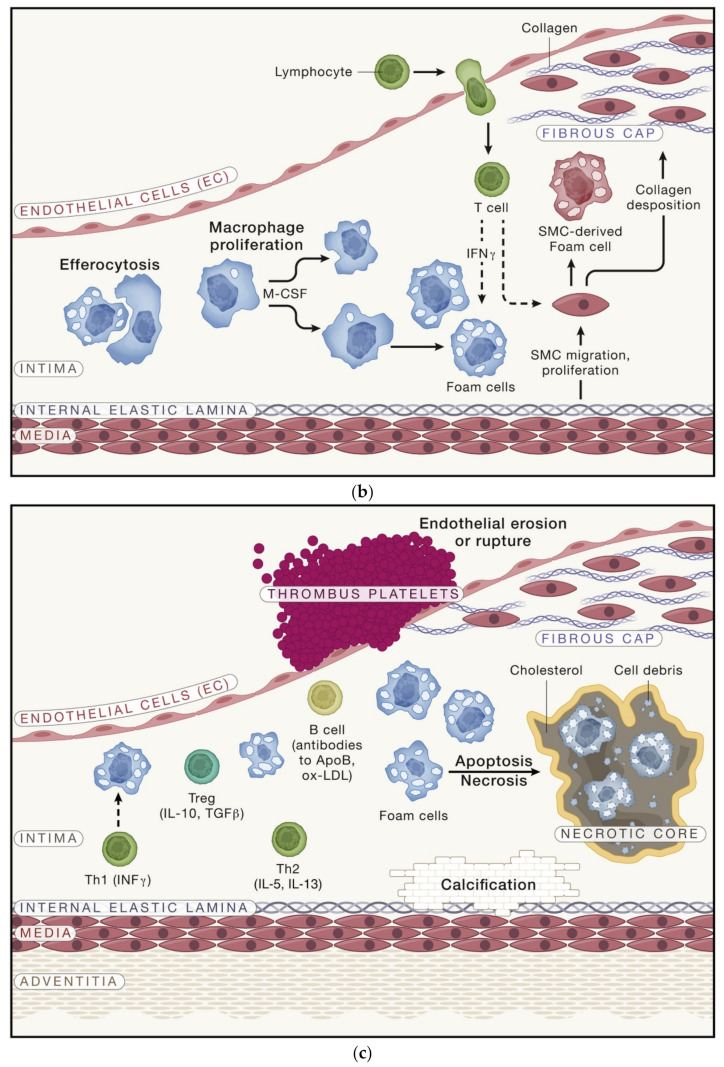
Stages of development of atherosclerotic lesions: (**a**) development of fatty streak lesions; (**b**) development of atherosclerosis lesions; (**c**) advanced atherosclerotic lesions (Figure 1 is taken from the work Björkegren and A.J. Lusis [9]).

In the present world, cardiovascular diseases (CVD) occupy a leading position in the structure of mortality [1,2]. Atherosclerosis plays an important role in the development of many CVDs: coronary heart disease, stroke, myocardial infarction, etc. [3,4]. Atherosclerosis is a chronic disease associated with inflammatory processes occurring primarily, with impaired lipid metabolism, arterial stiffness, the formation of foam cells and the blockage of the blood vessels [5,6,7,8]. The work of Björkegren and A.J. Lusis [9] describes in detail the growth of atherosclerotic lesions, cell types and molecular interactions involved in the initiation of lesions (Figure 1).

As it is shown in Figure 1a, under the conditions of disturbed blood flow, ECs become “leaky”, which promotes lipoprotein uptake and its entry into the intima. Atherosclerosis is initiated by the accumulation of certain plasma lipoproteins (particularly LDL and triglyceride-rich lipoprotein residues) in the intimal region of the vessel. In the intima, these lipoproteins are oxidized and aggregated, i.e., modified. This modification causes ECs to start expressing adhesion molecules (P-selectin, E-selectin, VACM1 and ICAM1) and chemotaxis molecules (CCR2 and CCR5) for monocytes. Monocytes bind to endothelial adhesion molecules and penetrate the intima, where they differentiate into macrophages in response to locally produced M-CSF and other cytokines. Macrophages engulf modified lipids, which can not be digested. As a result, macrophages form foamy cells.

Then, as depicted in Figure 1b, the modified lipids and foamy cells begin to accumulate, forming a focus of inflammation. Other leukocytes and T and B cells also enter this focus and interact with macrophages (promoting or slowing down the development of the lesion). Layers of smooth muscle cells (SMC) change from a contractile state to a proliferative one and migrate to the intima and endothelial area, forming a “fibrous cap” protecting the lesion from rupture. SMCs can differentiate into macrophage-like cells, which give rise to foam cells and bone-like cells, which deposit the calcium phosphate mineral.

At the next stage, the foam cells die off, forming necrotic nuclei consisting of cellular detritus and cholesterol, as presented in Figure 1 c. Calcification occurs in the intima or media. The lesions may rupture or the ECs may collapse, stimulating thrombus formation, which can lead to myocardial infarction or stroke. However, T cells affect the progression of the disease. T cells secrete IFNγ, which promotes plaque growth and instability. The regulatory T cells express anti-inflammatory cytokines (IL-10 and TGFβ), promote macrophage efferocytosis, and show a negative correlation with atherosclerosis. The TH2 cells express the anti-inflammatory cytokines IL-5 and IL-13. The B cells contribute to chronic inflammation in atherosclerosis.

Atherosclerosis, similar to other chronic diseases associated with the metabolic syndrome, is a multifactorial disease [10]. The development of atherosclerosis is associated with environmental exposure, genetic characteristics and functioning of the microbiota of the GIT of the consumer [11]. Commonly, the lifestyle factors include nutrition, physical activity, bad habits (smoking, alcohol abuse), climatic and geographical characteristics of living, and current health. The genetic features include heredity, the genes in which mutations affect the predisposition to metabolic disorders of the main nutrients ingested, impaired food behavior, leading to blockage of blood vessels, epigenetic factors, etc. The GIT microbiome is a collection of microorganisms (with common genetic material) inhabiting the GIT of the host organism [12]. The microbiota plays an important role in the functioning of the host organism, regulating the immune, endocrine and other systems [13]. Therefore, the microbiota is also involved in the development of atherosclerosis. So, the bacterial metabolites, such as SCFAs, choline, TMA, etc., affect the metabolism of bile acids, cholesterol, TMAO and influence inflammatory processes [14,15,16,17]. Accordingly, identification of the above predictors of the development of atherosclerosis contributes to the prevention and early treatment of a number of diseases.

**Figure 2:**
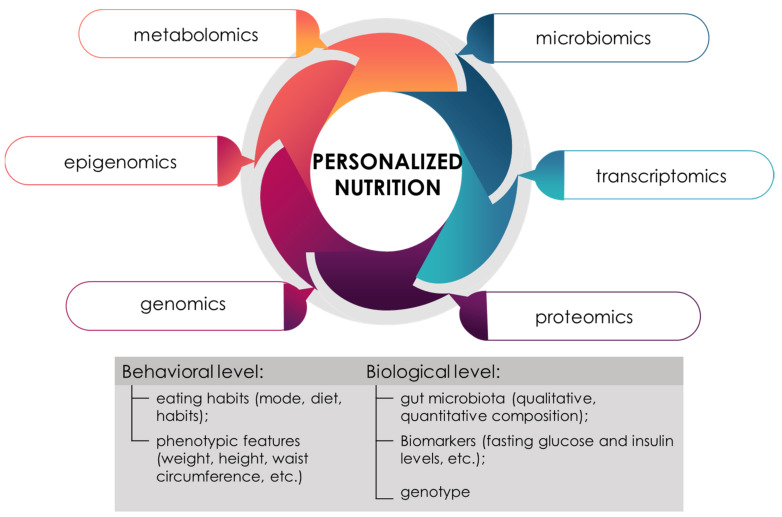
The importance of the influence of «omics» technology and the search for characteristics, the assessment of which is necessary for the formation of a personalized approach to nutrition.

Nutrition is an environmental factor that continuously affects the human body [12,18,19,20]. Eating disorders, unbalanced diet, vitamin deficiencies, excessive fatty foods, etc., disrupt the healthy state of the body. The concept of the “4Ps” (personalization, prediction, prevention and participativeness), based on the achievements of “omics” technology, under the concept of “good nutrition”, considers personalized, individual nutrition [21,22]. Personalized nutrition is nutrition that takes into account the personal characteristics of the consumer (characteristics of the phenotype, genotype, health status, lifestyle, a number of psycho–emotional factors, environmental, climatic conditions, religious, etc.), aimed at improving the healthy state of the body [23,24] (Figure 2).

The present work was aimed at the observation of the contributions related to the formation of personalized dietary recommendations for the prevention of atherosclerosis. The survey can help to understand what approaches exist today for the personalization of nutrition. We investigated what dietary recommendations are optimal for the prevention of atherosclerosis and how they can be modified for the consumer, for example, by enriching the diet with FF and dietary supplements. The comparative analysis reveals the most promising methods for evaluating the anti-atherosclerotic effect of BAS contained in food supplements, functional products, on model organisms, i.e., in preclinical studies.

## 2. Sources and Methods

The key questions were considered as follows:

1. How to fight against atherosclerosis with the help of nutrition and omics sciences.
2. What are the potential target markers that have contributed to the assessment of predisposition to the diagnosis of atherosclerosis?
3. What are the diets recommended for the prevention of atherosclerosis; what are the dietary supplements that exhibit an anti-atherosclerotic effect?

The following inclusion criteria were used in the work: search depth 27 years; language in Russian and English. To compile this systematic review, the authors observed published articles accounted in the following databases: PubMed (United States National Library of Medicine), Web of Science (Clarivate Analytics), Scopus (Elsevier), and the Russian Scientific Electronic Library eLIBRARY.RU. The results of intellectual activity posted in the databases of FIPS (Federal State Budgetary Institution “Federal Institute of Industrial Property”) and WIPO (World Intellectual Property Organization) were also considered. The main attention was paid to the data obtained from clinical, preclinical studies aimed at studying dietary interventions and dietary supplements, the genetic features affecting nutrition, and the development of atherosclerosis. The following keywords were used in the search: atherosclerosis, cardiovascular diseases, personalized nutrition, personalized nutrition and atherosclerosis, nutritional genetics, nutritional genetics and atherosclerosis, nutrigenetics, nutrigenetics and atherosclerosis, genes and atherosclerosis, SNP and atherosclerosis, nutrigenetic testing, epigenetics of nutrition, epigenetics of nutrition and atherosclerosis, chronological nutrition, chronological nutrition and atherosclerosis, nutrigenomics, nutrigenomics and atherosclerosis, dietary models and atherosclerosis, ketogenic diet, ketogenic diet and atherosclerosis, DASH-diet, DASH-diet and atherosclerosis, Mediterranean diet, Mediterranean diet and atherosclerosis, functional foods and atherosclerosis, biologically active substances and atherosclerosis, Model organisms and atherosclerosis, microbiota of the GIT and atherosclerosis, probiotics and atherosclerosis, recommendations for the prevention of atherosclerosis.

The authors reviewed all the publications obtained as a result of the search and selected the most important contributions to compile this review. Thus, 317 publications and 25 patents were accounted in the work.

## 3. Results and Discussion

The personalization of nutrition covers an extensive set of factors affecting health (in particular, the development of atherosclerosis) associated with eating [25,26,27]. Nutrition genetics, the influence of nutrition on the state of the gastrointestinal microbiota, provides a huge contribution to the development of atherosclerosis [28]. Nutrition genetics is a science that studies the relationship between nutrition and the genetic characteristics affecting the health of consumers [19]. Nutrition genetics is divided into nutrigenetics, nutrigenomics and epigenetics [23,24]. That is, nutrition genetics includes classical studies of human nutrition, the interaction of genes and diet, studies on *in vitro* and *in vivo* models using omics technologies [23]. In the work of N.E.A Gaboon, the components of nutrition genetics are highlighted [27]:

1. Diet is an important risk factor for the development of many diseases.
2. Food components directly or indirectly affect the genome (expression of genes, proteins).
3. The effect of the diet on the health of the consumer depends on the genetic characteristics of the consumer.
4. Genes whose functioning is influenced by food components are risk factors for the occurrence, progression, and severity of a number of chronic diseases.
5. Personal nutrition has a preventive, therapeutic focus.

**Figure 3:**
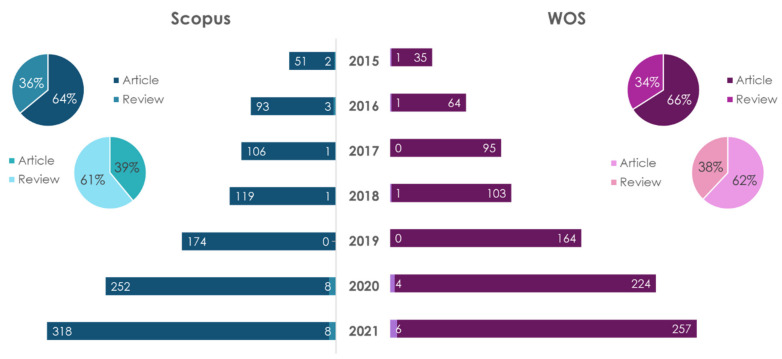
Statistical results of the search in the Scopus/WOS databases for the keywords “personalized nutrition” (dark color) and “personalized nutrition and atherosclerosis” (light color).

**Figure 4:**
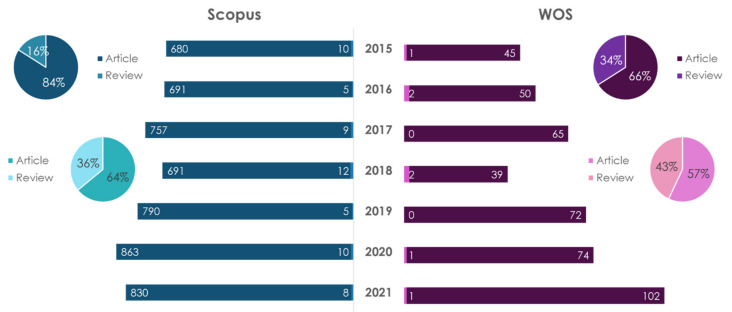
Statistical results of the search in the Scopus/WOS databases by the keywords “nutritional genetics” (dark color) and “nutritional genetics and atherosclerosis” (light color).

The dynamics of publications in recent years on the subject of personal nutrition (nutrition genetics) for the prevention of atherosclerosis are shown in Figure 3 and Figure 4.

The number of scientific papers in this research field is growing every year. A search for “personalized nutrition” showed that, regardless of the database, the number of research-type publications (articles) dominated the number of review articles. In the Scopus database, the number of articles is 1.78 times more than reviews, and in WOS it was 1.94 times. The largest number of publications on this topic, regardless of the database and type of publication, belongs to USA. Thus, in the period of 2015–2021, 211 articles and 115 reviews were accounted in the Scopus database, and 189 articles and 81 reviews were accounted for in the WOS database.

A search for “personalized nutrition and atherosclerosis” showed that in the Scopus database the number of reviews exceeded the number of articles by 1.75 times, and in the WOS database, the number of article exceeded the number of reviews by 1.6 times. The largest number of publications on this topic, according to the articles, belongs to the USA (four works in Scopus and four in WOS); according to reviews in Scopus—Italy (four articles)—and in WOS—Canada (one publication).

The number of scientific papers in this research field is growing every year (the period under review is from 2015 to 2021). A search for “nutritional genetics” and “nutritional genetics and atherosclerosis” showed that, regardless of the database, the number of publications (article type) prevails the number of reviews. A search for “nutritional genetics” indicated that, regardless of the type of publication and database, the USA is the leader in the number of papers (1542 articles in Scopus and 99 articles in WOS; 269 reviews in Scopus and 68 reviews in WOS). A search for «nutritional genetics and atherosclerosis» showed that USA is the leader in the number of publications in Scopus (17 publications of the article type, 6 review articles). In WOS, Brazil is the leader in the number of published articles, and the United States is the leader in review articles.

### 3.1. Nutrigenetics

**Figure 5:**
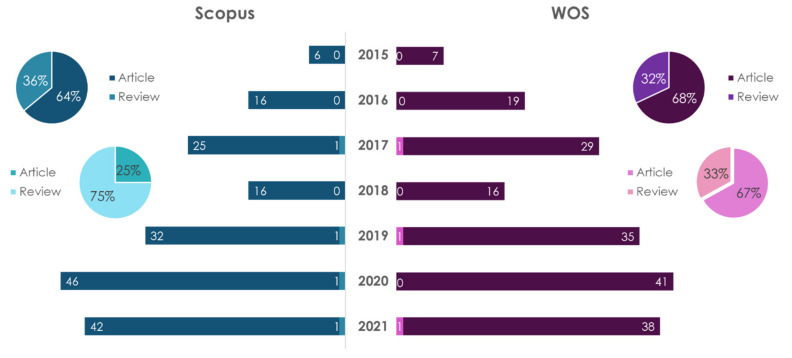
Statistical results of the search in Scopus/WOS databases by keywords “nutrigenetics” (dark color) and “nutrigenetics and atherosclerosis” (light color).

Nutrigenetics studies the influence of genetic features (mutations, the presence of polymorphisms, etc.,) on the metabolism of nutrients and the body’s response to them [29,30,31]. The dynamics of publications on the topic of nutrigenetics is reflected in Figure 5.

The obtained data show that in 2018 there was a decline in publications on this topic; namely, in 2020 the number of papers increased by 2.9 times when compared to 2018 and 7.7 times when compared to 2015. The leader in the number of publications on “nutrigenetics”, regardless of the database and type of publication, is the USA (31 articles and 14 reviews in Scopus; 7 articles and 13 reviews in WOS) for the period of 2015–2021. The leader in the number of publications on “nutrigenetics and atherosclerosis” for articles is a collaboration of authors from USA and Brazil. The leader in the number of articles on “nutrigenetics and atherosclerosis” in the database Scopus is Italy, and in the database WOS the leader is the USA.

**Figure 6:**
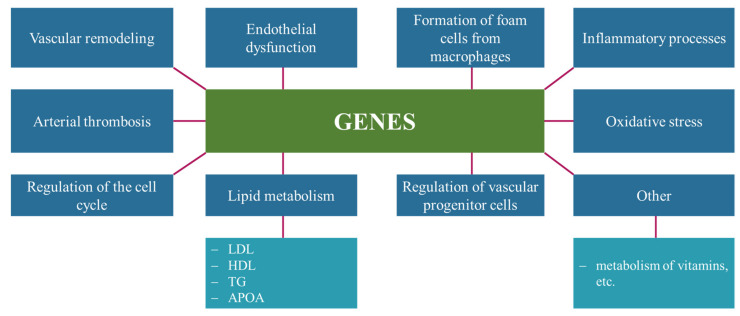
Genes affecting the development of atherosclerosis.

It is known that the number of SNPs plays a key role in the development of atherosclerosis [19,20,32]. A list of genes affecting the development of atherosclerosis is presented in Figure 6 [33,34].

In nutrigenetic studies, it was shown that consumers with certain genotypes (SNP) respond better/worse to diet, and it explains the importance of nutrigenetic testing for the formation of personalized dietary recommendations [24,35]. Atherosclerosis is a multifactorial disease, and its development involves genes affecting inflammatory processes (for example, interleukins: *IL-1*, *IL-1Ra*, *IL-6*, *IL-10*; cytokines: *TNF*-α, *TNF*-receptor, *LT-α*; adhesion molecules: selectins, ICAM-I, VCAM-I, PECAM; chemokines: *CX3CR1*, *CCR5*, *CCR2*, *CXCL12*, *MCP-1*, etc. [36,37]), the formation of foam cells (*PCSK9*, *LOX-1*, *ALDH2*, etc. [38,39]), the accumulation of cholesterol by macrophages (*IL7R*, *IL7*, *TIGIT*, *IL-8*, *F2RL1*, *EIF2AK3*, *TSPYL2*, *ANXA1*, *DUSP1* и *IL15* [40]), and the accumulation of the protein MMP12 (SNP next to the gene *MMP12* on the chromosome 11q22.3) [41] on the body’s antioxidant defense system (*SOD*, *CAT*, *GPX1*, etc. [42]) and cholesterol metabolism (*ABCA1*, *ABCG1*, *APOA*, *APOB*, *APOE*, *CETP*, *LIPC*, etc.), etc. [35]. It is promising to consider genes that are able to affect a person’s eating behavior (eating habits), for example: [10,24,43]:

*CD36*, polymorphisms (rs1761667, rs1049654, rs10499859, rs1527483, rs3211956) which affect taste perception (sensitivity) to fats in food [44,45,46,47,48];

*FTO* (rs9939609), GHRL (rs34911341), LEPR (rs1137101), polymorphisms which affect the amount of food consumed and the feeling of satiety [49,50,51,52,53,54,55];

*MC4R*, the polymorphism (rs17782313) which affects the regulation of appetite and the feeling of satiety [49,56,57,58];

*GLUT2* (rs5400) and TAS1R2 (rs12033832), polymorphisms of which affect the taste perception of sweet [59,60,61,62];

*TAS2R38*, polymorphism (rs1726866); these polymorphisms affect the taste perception of bitter [63,64];

*ADD1* (rs4961) and CYP11B2 (rs1799998); these polymorphisms affect the consumption of table salt [65,66,67,68];

*DRD2*, the polymorphism rs1800497 affects the synthesis of dopamine and the development of dependence on the “jamming” of stress [69,70,71];

*CYP1A2* and hypertonic reaction to caffeine consumption [24,72];

These genes and their influence on eating behavior were considered in more detail in an early study [73].

A large list of genetic features (mutations) that affect the development of diseases prompts the question: “What number of genes is optimal in order to assess the risks of developing the disease and prescribe a personal preventive diet?” The answer for this question is not clear and the number of genes studied varies in different works. However, mostly in genetic studies, the influence of SNP genes affecting the metabolism of lipids, carbohydrates, antioxidants, vitamins, and the effect on taste preferences (eating behavior) is evaluated.

So, in Nutrigenomix Inc (Canada), about 70 genetic markers were investigated [74]. 134 genes were analyzed by 3 × 4 Genetics (South Africa) [75]. In the company “Mapmygenome” (India), the screening of over 700,000 genetic markers across the human genome was carried out [76]. In the company “GenoPalate Inc” (USA), over 100 genetic markers were observed [77]. In the company «HELIX» (Russia), in its nutrigenetic testing, the analysis of 80 genes was implemented [78]. In the company “Center for New Medical Technologies” (Novosibirsk, Russia), 14 genes were considered [79]. Fifty-two genes were analyzed by the company “MyGenetics” (Novosibirsk, Russia) [80]. In the company “Genotek” (Moscow, Russia), more than 5000 genes, 14,000 mutations and 4000 hereditary diseases [81] etc., were diagnosed. In general, companies that make their personalized recommendations based on nutrigenetic testing take into account only one or a few gene polymorphisms, which leads to incomplete health reporting and misconceptions [82].

When using nutrigenetic testing, it is important to avoid the misuse of genetic information and to protect basic human rights to safety, privacy, etc. [24]. It is needed to keep in mind that personal dietary recommendations based on nutrigenetic testing must meet a number of credibility criteria. Thus, all studies examining gene–diet interactions should follow the recommendations of STREGA (“Strengthening Genetic Association Research Reporting”) [83], describing the algorithm for selecting participants for the experiment and errors that may occur during the experiment. In the work of K. A. Grimaldi [21], an algorithm was proposed for assessing the scientific validity of gene–nutrition interactions:

1. Analytical validity, i.e., a measure of the accuracy of genotyping (accredited laboratories), must be observed.
2. Scientific validity of the data, i.e., convincing evidence of how a particular gene (SNP) affects a particular condition. The following criteria have been proposed to assess validity: study quality rating (randomized, placebo-controlled and blinded, prospective and retrospective approach, etc.), gene–nutrition interactions (direct, intermediate or indirect phenotype), the nature of the genetic variants (causal, associated, but with unknown function, etc.), biological plausibility (high, medium, low, unknown), assessment of scientific validity of the gene–diet interaction (conclusive, likely, possible and unproven).
3. Compliance with ethical, legal and social norms when conducting, interpreting and prescribing a personal diet to the consumer [84,85].

Well-written recommendations (correct interpretation of the results) require highly qualified specialists in the fields of medicine, genetics and nutrition (nutritionists, etc.) [24]. That is, in the formation of personalized nutrition, the collaboration of specialists of various profiles is necessary. In the work of D. Noland [86], standard practices and standards of practice were presented for registered dietitians and nutritionists, divided into three levels of practice: competent, experienced and expert. This reflects the principles of integrative and functional medicine with an emphasis on nutrition. For the formation of individual preventive FF and biologically active BAA, food production technologists, analytical chemists, etc., are needed.

In addition to SNP, other genetic traits influence the development of atherosclerosis. Of particular interest is the relationship between nutrition and telomere length (repetitive, terminal sections of chromosomes) [20]. Telomere shortening leads to cell aging, and since there is a significant shortening of telomeres in endothelial cells in the areas of atherosclerotic lesions, the pathogenesis of atherosclerosis is also associated with aging [87]. Telomere length is affected by a number of factors: diet, smoking, alcohol and metabolic syndrome. The effect of nutritional quality on telomere shortening was examined by A. Ojeda-Rodríguez [88]. As it was shown, following an MD reduces the risk of telomere shortening. It is known that folic and nicotinic acid deficiency increased oxidative stress and telomere dysfunction. Methyl donors (folate, vitamin B11, choline, methionine) are involved in maintaining cytosine methylation. Defects in DNA methylation cause an excessive elongation of telomeres and homologous recombination between telomeres and their fusion [32]. Epigenetic factors also influence the pathogenesis of atherosclerosis [20,89].

#### 3.1.1. Epigenetics and Nutrition

**Figure 7:**
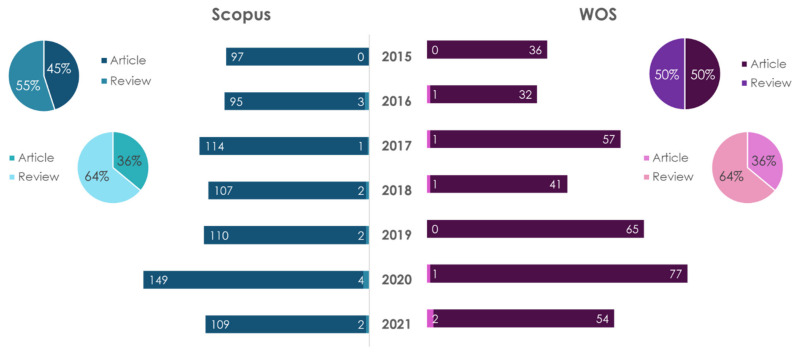
Statistical results of the search in Scopus/WOS databases by the keywords “epigenetics of nutrition” (dark color) and “epigenetics of nutrition and atherosclerosis” (light color).

A consensus is reached among scientists that atherosclerosis is an epigenetic disease [89,90,91,92,93]. There is a relationship between increased DNA methylation and CVD [93]. The dynamics of publications in this research field from 2015 to 2021 are shown in Figure 7.

It is seen in Figure 7 that, in 2020, the maximum number of publications appears on this topic. The number of review on “epigenetics of nutrition” in the Scopus database is 1.22 times higher than that in the WOS database. The number of scientific publications (article type) is the same in all databases. On the subject of “epigenetics of nutrition and atherosclerosis”, regardless of the database, the number of reviews is higher than the number of articles: in the Scopus database, the number of reviews is 1.80 times higher than the number of articles, and, in the WOS database—by 2.00 times.

The leader in the number of publications on “epigenetics of nutrition”, regardless of the database and type of publication, is the USA (138 articles and 150 reviews in Scopus; 86 articles and 59 reviews in WOS) over the period of 2015–2021. The leader in the number of publications on “epigenetics of nutrition and atherosclerosis” for articles in the Scopus database is Belgium, and, in WOS—the USA. According to reviews in the Scopus database, the leader is France, and in WOS—the United Kingdom.

Some food components are known to have epigenetic effects, including DNA methylation (methionine, folic acid, B vitamins [20]), histone acetylation/deacetylation and small non-coding RNA actions. In the work [94], the data are presented on the effect of DNA methylation on the development of CVD (model subject—mice): it was found that the hypomethylation of DNA is associated with decreased expression of DNMTs and MTHFR genes. In APOE-knockout mice, there is a trend to promote specific changes in DNA methylation, occurring in peripheral blood leukocytes [94]. In [89], the genes are listed that are associated with the development of atherosclerosis and are partially regulated by DNA methylation: eNOS, iNOS, FADS2, estrogen receptor alpha and beta genes (ERα [95], *ERβ*), *EC-SOD*, etc. It was shown that low LINE-1 methylation predicted CVD risk [96].

To select individual BAS that can influence the epigenetic characteristics of the consumer, additional research is needed to study the influence of epigenetic factors on the development of CVD and find new biomarkers of CVD and new models to study the epigenetic effects of BAS, etc. [90].

#### 3.1.2. Chronological Nutrition

**Figure 8:**
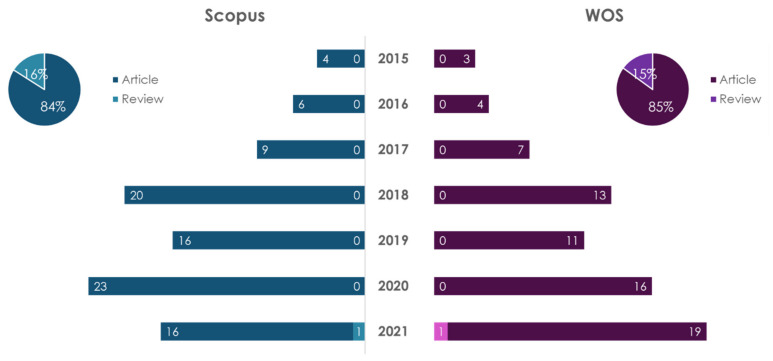
Statistical results of the search in Scopus/WOS databases by the keywords “chronological nutrition” (dark color) and “chronological nutrition and atherosclerosis” (light color).

Circadian rhythms play a specific role in the development of metabolic diseases [97]. The results of the statistical analysis of publications on this topic for the period from 2015 to 2021 are shown in Figure 8.

The obtained data show that the number of publications was growing until 2020. In 2021, there was a decline in publication activity in this field. Regardless of the database, a search for “chronological nutrition” showed that the number of reviews exceeds the number of articles: in the Scopus database by 5.3 times, in the WOS database by 5.63 times. A search for “chronological nutrition and atherosclerosis” yielded only two articles (one article in Scopus, one article in WOS by author teams from Switzerland). The leader in the number of publications on “epigenetics of nutrition”, regardless of the database and type of publication, is the USA (138 articles and 150 reviews in Scopus; 86 articles and 59 reviews in WOS) for the period of 2015–2021. The leader in the number of publications on “chronological nutrition”, according to articles in all reviewed databases is the USA and according to reviews it is Italy.

Chrono-nutrition is a research field, where the effects of time-limited nutrition on cellular physiology and metabolism are investigated. Chrono-nutrition and nutritional genetics study common themes—the influence of genes (including circadian genes) on metabolism and the healthy body. In the work [97], it is pointed out that the circadian system regulates the functions of the cardiovascular system [98]. There is evidence that circadian rhythms, consisting of a network of genes, are associated with metabolic disorders and with the health of the body. Circadian rhythms are also modulated by epigenetics (influence of circadian microRNAs) [99]. In [100], it is shown that endothelial function, clot formation and BP are regulated by the circadian clock; myocardial infarction, arrhythmia and heart failure are associated with disruption of circadian rhythms. It was discovered that circadian clock dysfunction leads to the development of atherosclerosis [101]. Consequently, personal nutrition combined with chrono-nutrition can contribute to the prevention of metabolic syndrome [102]. However, circadian regulation is a relatively new field that requires a lot of research.

### 3.2. Nutrigenomics

**Figure 9:**
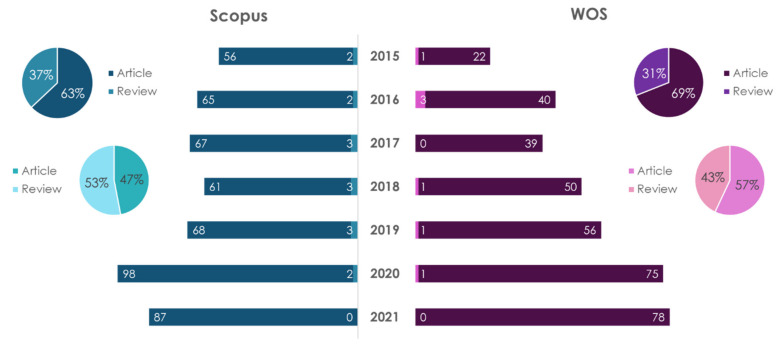
Statistical results of the search in Scopus/WOS databases by the keywords “nutrigenomics” (dark color) and “nutrigenomics and atherosclerosis” (light color).

Nutrigenomics studies how certain foods (nutrients) affect gene expression and disease development [18,29,30,31]. The technologies used in genomic research include transcriptomics, proteomics and metabolomics [32]. The dynamics of publications in this research direction are reflected in Figure 9.

The number of scientific papers on this topic is growing every year. A search for “nutrigenomics” showed that regardless of the database, the number of research-type publications (article) prevails over the number of reviews. The largest number of publications on this topic, regardless of the database or the type of publication, belongs to the USA (in the period of 2015–2021, 102 articles and 55 reviews in Scopus, 82 articles and 29 reviews in WOS). A search for “nutrigenomics and atherosclerosis” indicated that, in the Scopus database, the number of reviews exceeds the number of articles by 1.33 times, and, in the WOS database, the number of articles exceeded the number of reviews by 1.14 times. The largest number of publications on this topic in the Scopus database (reviews and articles) belongs to Italy. In the WOS database, Italy is the leader for articles, and Canada is the leader for reviews.

It is known that nutrition plays an important role in the development of atherosclerosis [103]. For example, diets high in calories and fats and the excessive consumption of animal proteins are risk factors for the development of CVD [104,105]. Oppositely, diets including a moderate consumption of dairy products, fortification with fruits, vegetables, legumes, nuts, a source of animal protein, e.g., poultry and fish, a minimum content of trans fats, carbohydrates, red meat, etc., [106] are effective means for the prevention of CVD. In [107], ten fruits were revealed, the use of which has a beneficial effect on the cardiovascular system. These fruits include apples, avocado, grapes, mango, orange, kiwi, pomegranate, papaya, pineapple and watermelon. These fruits improve the endothelial function of blood vessels, modulate BP, lower cholesterol, and reduce the formation of blood clots and oxidative stress [108].

#### 3.2.1. Dietary Models for the Prevention of Atherosclerosis

There are various dietary models aimed at normalizing the body metabolism. These diets are mainly aimed at reducing BMI [109], for example, low-carbohydrate (ketogenic), LGI diets, the MD, the DASH-diet. These diets are also suitable for the prevention of atherosclerosis, since it has been proven that CVD are closely linked to obesity [110].

**Figure 10:**
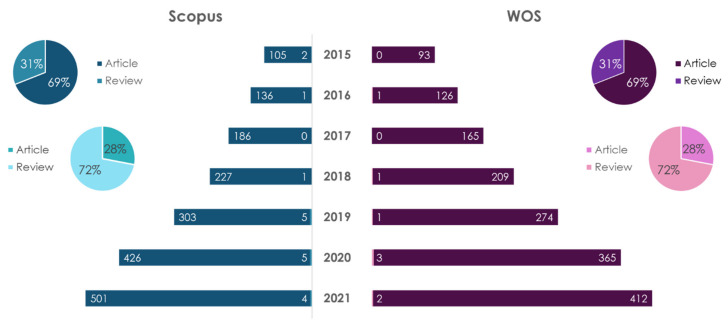
Statistical results of the search in Scopus/WOS databases by the keywords “ketogenic diet” (dark color) and “ketogenic diet and atherosclerosis” (light color).

**KD**—diets with minimal carbohydrate intake, moderate protein intake and increased fat intake [111,112]. The dynamics of publications on this topic from 2015 to 2021 are shown in Figure 10.

As it is evident in Figure 10, the number of publications on these topics is increasing. A search for “ketogenic diet” showed that the number of articles in the Scopus and WOS databases is 2.60 times greater than the number of reviews. A search for “ketogenic diet and atherosclerosis” revealed that the number of reviews in the Scopus database is 2.6 times greater than the number of articles, and the number of articles in the WOS database is 3.0 times greater than the number of reviews. The leader in the number of publications on “ketogenic diet”, regardless of the database and type of publication, is the USA (528 articles and 227 reviews in Scopus; 492 articles and 198 reviews in WOS) for the period of 2015–2021. The leader in the number of publications on “ketogenic diet and atherosclerosis” by articles in the considered databases is also the USA. The leader in the number of reviews in Scopus is the USA, and in WOS—France.

The peculiarity of the KD is that the body gets energy not from carbohydrates but from fats. This diet increases the LDL level; however, it also increases the HDL level that helps to reduce the TG level, total cholesterol to HDL ratio and *APOB/APOA1* ratio [113]. The effects of AI and a low-fat diet on weight loss and lipid levels with overweight people were evaluated [114]. A significant decrease in LDL-C was observed in the group following a low-fat diet. The effect of a KD with a high content of vegetable proteins from gluten, soy, nuts, fruits, vegetables, cereals and vegetable oils was estimated for weight loss and LDL concentration, as compared with a high-carbohydrate diet based on low-fat dairy and whole grain products [115]. The results showed that weight loss was similar in both groups, but people on the KD had the greatest reductions in LDL, total cholesterol to HDL-C and *APOB/APOA1*, and systolic and diastolic BP. In [116], it was also shown that a diet with a high protein content and a low carbohydrate content is efficient for reducing fat mass and it leads to a steady decrease in TG levels and an increase in HDL compared to a diet with a high carbohydrate content. The diets of the US National Educational Program on cholesterol (replacement of saturated fats with carbohydrates) were compared with a diet in which there was a replacement of fats with proteins with monounsaturated fats (proposed by the authors) [117]. As a result, the diet proposed by the authors led to the greatest reduction in cholesterol levels and was not inferior in terms of BMI reduction.

The presented data show that the KD is acceptable for reducing BMI (i.e., eliminates one risk factor—obesity) and blood lipid levels. However, in addition to the emphasis on reducing carbohydrates for the prevention of atherosclerosis (and other CVD), it is necessary to consider the types of proteins included in the diet. The effect of animal and vegetable protein consumption on cardiometabolic risk factors was observed in [118]. As a result, the consumption of plant-based proteins and some animal proteins (poultry, fish, unprocessed red meat with a low content of saturated fat and low-fat dairy products) can have a positive effect on risk factors; that is, these proteins are optimally included in dietary recommendations for the prevention of atherosclerosis. Animal proteins, on the contrary, need to be limited.

**Figure 11:**
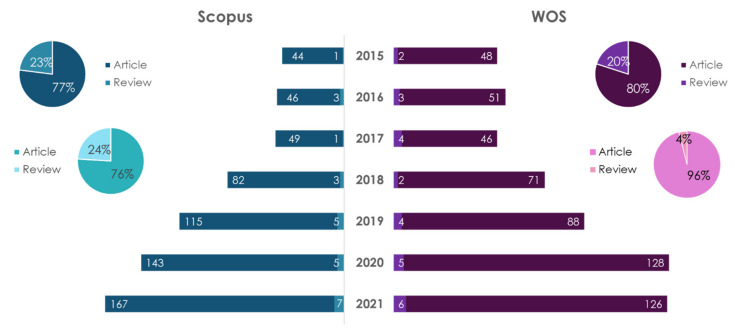
Statistical results of the search in Scopus/WOS databases by the keywords “DASH-diet” (dark color) and “DASH-diet and atherosclerosis” (light color).

**DASH-diet** is a diet characterized by eating/limiting the following eight key foods: eating fruits, vegetables, whole grains, nuts and beans, and low-fat dairy products, and limiting the consumption of red and processed meat, sweetened beverages and salt [105,119]. The diet is characterized by a low content of saturated fats, dietary cholesterol, salt (sodium) and a high content of dietary fiber, potassium and calcium [120]. The DASH-diet is associated with normalization of the BP, glucose–insulin homeostasis, reduced cholesterol levels, functioning of the gastrointestinal microbiota, and a decrease in BMI [121]. It was obtained that compliance with the DASH-diet correlates with low levels of C-reactive protein in plasma and interleukin 6, i.e., affects inflammatory processes [122]. The dynamics of publications on this topic from 2015 to 2021 are shown in Figure 11.

The diagrams show that the number of publications on these topics is growing. A search for “DASH-diet” yielded that the number of articles in the Scopus database is 3.31 times higher than the number of reviews. The number of articles in the WOS database is 4.12 times higher than the number of reviews. A search for “DASH-diet and atherosclerosis” indicates that the number of articles in the Scopus database is 3.17 times higher than the number of reviews. The number of articles in the WOS database is 25 times higher than the number of reviews. The leader in the number of publications on “DASH-diet”, regardless of the database and type of publication, is the USA (280 articles and 59 reviews in Scopus; 271 articles and 32 reviews in WOS) over the period of 2015–2021. The leader in the number of publications on “DASH-diet and atherosclerosis” by the type of articles in the databases under consideration is the USA. As regards the reviews in the Scopus database, the USA is the leader, and in WOS—the United Kingdom.

**Figure 12:**
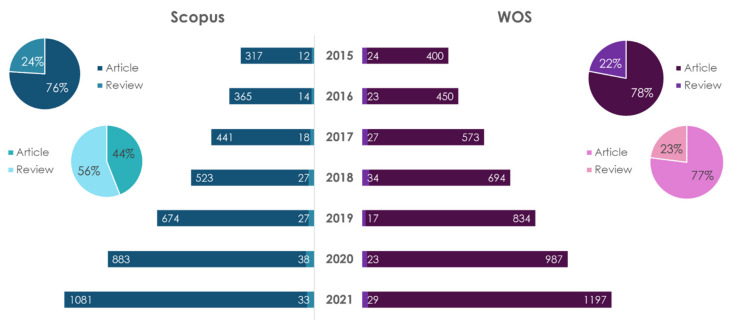
Statistical results of the search in Scopus/WOS databases by the keywords “Mediterranean diet” (dark color) and «Mediterranean diet and atherosclerosis» (light color).

One of the most popular diets for the prevention of metabolic syndrome, in particular CVD, is the MD [106,123,124]. The dynamics of publications on this topic from 2015 to 2021 is shown in Figure 12.

As it is seen in Figure 12, the number of publications on these topics is drastically growing. A search for “Mediterranean diet” showed that the number of articles in the Scopus database is 3.2 times higher than the number of reviews. The number of articles in the WOS database is 3.5 times higher than the number of reviews. A search for “Mediterranean diet and atherosclerosis” resulted that there are 1.25 times more reviews in the Scopus database than the number of articles. In the WOS database, the number of articles is 3.3 times higher than the number of reviews. The leader in the number of publications on the “Mediterranean diet” by the type of article in the databases under consideration is Spain. As to reviews accounted for in the Scopus database, the leader is USA, and in WOS—Italy. The leader in the number of publications on “Mediterranean diet and atherosclerosis” by the type of article in the databases under consideration is USA. According to the reviews accounted in the databases, Italy is the leader.

The **MD** is a diet common in the Mediterranean countries. The cardioprotective effect of the diet is based on the content of nutrients exhibiting antioxidant properties, epigenetic, and genetic effects (it is known that the polymorphism rs1801282 of the *PPARγ2* gene affects telomere homeostasis in people with a high risk of CVD, and compliance with MD prevents telomere shortening [125,126,127]) [64,128,129]. It is known that MD has a positive effect on the functioning of the gastrointestinal microbiota [12,13,20,31]. The dietary factors in MD are described in [123]—they include a high intake of plant foods (it is important that the diet is dominated by fresh fruits and vegetables), cereals and whole grain bread, beans, nuts and seeds; the source of lipids is olive oil; moderate consumption of milk and dairy products; a small amount of red meat and more fish; a small/moderate amount of red wine [106,130,131]. MD also includes FF, that is, foods that contain a number of BAS, for example, fermented dairy products [131,132]—yogurt, cheese, nuts, vegetables, fruits—as well as various spices and herbs (plants).

In [133], the following diets were mentioned, as possibly being related to the prevention of CVD, as some of the foods in them show cardioprotective potential.

The **Japanese diet** is a traditional diet that promotes longevity. The diet is rich in fish, seaweed, soy products, fruits and vegetables, rice, Japanese pickles, green tea [134].

The **Scandinavian (Nordic) diet** is a diet that emphasizes traditional, local and seasonal foods from Scandinavian countries, including oily fish (salmon and mackerel), vegetables, root vegetables, legumes, fruits, berries, and whole-grain cereals (oats, rye, and barley). The Nordic diet is rich in dietary fiber and low in sugar and salt [120].

The **Portfolio Diet** is a predominantly plant-based, vegan diet low in saturated fat and cholesterol. The diet is designed to lower total cholesterol and LDL [120,135]. That is, it is a vegetarian diet that includes the consumption of FF.

**Vegan/vegetarian diets** are diets that exclude meat, fish, eggs, dairy products and honey. Diets are high in dietary fiber and low in fat, omega-3 fatty acids, iron and vitamin B12 [120]. Individuals following this diet have low levels of BP, cholesterol, BMI [136]. A review implemented by C.S. Kwok looked at the effects of a vegetarian diet on CVD [136]. The diet was shown to be potentially associated with reduced risk of coronary heart disease. In [137], the effect of vegan diet on CVD considered, but clinically reliable data on the relationship between this diet and CVD were not found.

As it was shown in [138], the diets with a high glycemic load and glycemic index are associated with the risk of CVD. The effect of a diet with an LGI on body weight, carbohydrate and lipid metabolism in comparison with a diet with a high glycemic index were evaluated in [139]. As a result, for people adhering to a diet with an LGI their body weight was significantly reduced, and there was a decrease in feelings of hunger before lunch and dinner. There was no increase in insulin sensitivity, and lower levels of total cholesterol, LDL were observed. In [140], the effects of four different diets on weight loss and CVD risk reduction were evaluated. Diet 1—high carbohydrate, medium protein and foods with a high glycemic index. Diet 2—high carbohydrate, medium protein and LGI foods. Diet 3—high protein low carbohydrates and foods with a high glycemic index. Diet 4—high protein low carbohydrate and LGI foods. As a result, Diet 1 showed the slowest decrease in BMI, Diet 2 showed the greatest loss of BMI and a decrease in LDL levels. Diet 3 resulted in an increase in total cholesterol and LDL levels. Diet 4 resulted in a decrease in total cholesterol and LDL levels.

The effect of a gluten-free diet on the primary prevention of CVD was revealed in [141]. As a result, data were obtained showing that there was no clear correlation between the gluten-free diet and CVD mortality; that is, gluten consumption was not associated with the development and prevention of myocardial infarction, with changes in BP, LDL levels and BMI.

In addition to including/excluding/restricting certain foods from the diet, interval eating is of interest. Intermittent or **intermittent fasting**—a type of diet that limits the time you eat, such as fasting for one or two days a week, fasting every other day, or eating only at certain hours and fasting for at least 12 h each day. In [142], the effect of intermittent fasting on the prevention of CVD was evaluated. As a result, to date, there are no data confirming the clinical significance of intermittent fasting on CVD. Further studies in this direction are needed.

There is a considerable debate as to which diet is best for individual patients [118]. This issue must be decided individually, based on the existing risk factors, the financial and moral state of the consumer, etc. It is important that following any of the diets that reduce/eliminate the intake of certain nutrients is not a risk factor. For example, people on a KD are characterized by a lack of fiber, minerals, and iron, which negatively affects the functioning of the gastrointestinal microbiota, because the availability of a number of carbohydrates necessary for representatives of the intestinal microbiota is reduced. Therefore, they need an additional intake of foods rich in fiber (nuts, broccoli, cauliflower, berries, etc.).

#### 3.2.2. BAS, FF with Cardioprotective Activity

Groups of foods that are recommended for preventing CVD consist of fruits and vegetables, olive oil, nuts, wine, and other fermented alcoholic beverages. The benefits of eating these products are due to their content of bioactive compounds: antioxidants (polyphenols, vitamins), dietary fiber, trace elements, fatty acids (omega-3), etc. [121]. Among these products (in addition to fruits and vegetables), plants are a promising source of BAS. In Table 1, a list of plant-based cardioprotectors is presented [143].

Some of the above BAS are plant metabolites often used in traditional medicine in different countries. The cardioprotective effect of these substances is based on their antioxidant action (protect lipids from oxidation), the ability to inhibit the expression of a number of genes (adhesion, inflammation, etc.), and regulate the composition and metabolites of the GIT microbiota. The source of these cardioprotective substances can be animal and plant raw materials (Table 2).

The anti-atherosclerotic effect of these BAS is also expressed in the antioxidant action, in the ability to inhibit the expression of genes involved in the pathogenesis of atherosclerosis and in the anti-inflammatory action. The BAS (Table 3) that affect epigenetic mechanisms involved in the development of atherosclerosis were identified in [12,91].

**Figure 13:**
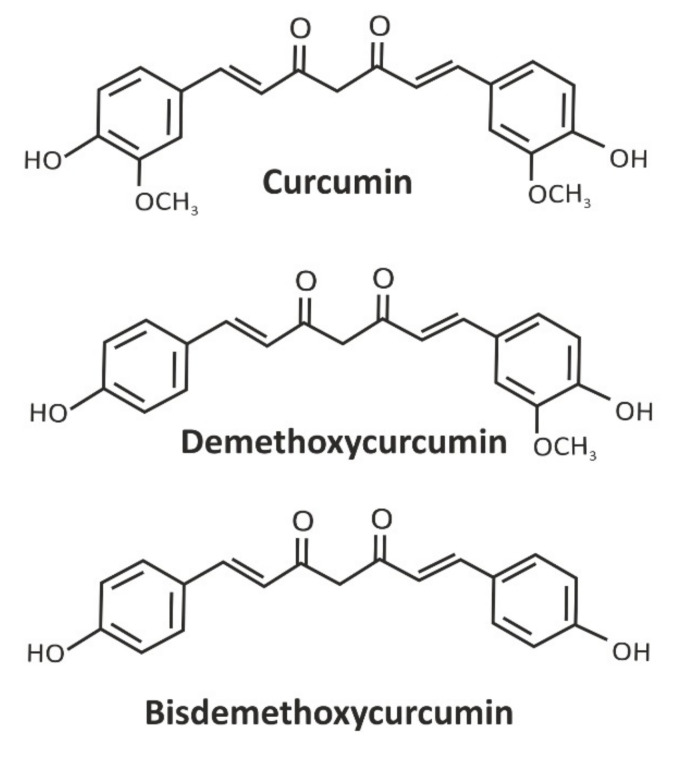
Major metabolites of *Curcuma longa*.

Curcumin is of particular interest as an epigenetic modifier. Curcumin is a plant polyphenol whose source is the plant turmeric (*Curcuma longa*). The main metabolites of turmeric are shown in Figure 13 [217].

Curcuma and curcumin have been widely and safely used for hundreds of years as a natural food coloring. Preclinical studies showed that curcumin exhibits a wide range of biological activities: anti-inflammatory, anti-cancer, antioxidant and other activities [218,219,220]. Studies show that turmeric metabolites have antiproliferative and proapoptotic effects on pancreatic cancer cells [221], prostate cancer cells [222] and cell lines of malignant mesothelioma [223]. Curcumin not only effectively removes active oxygen, but also activates elements of the antioxidant response by inhibiting lipid peroxidation [224]. Interestingly, curcumin inhibits the production of reactive oxygen species at low concentrations, but induces the production of reactive oxygen species at high concentrations [225]. Depending on the cell type, curcumin can exhibit both antioxidant and pro-oxidant effects [226]. Curcumin increases glioblastoma cell death by inhibiting the signaling pathway of PI3K/Akt/mTOR [227].

**Figure 14:**
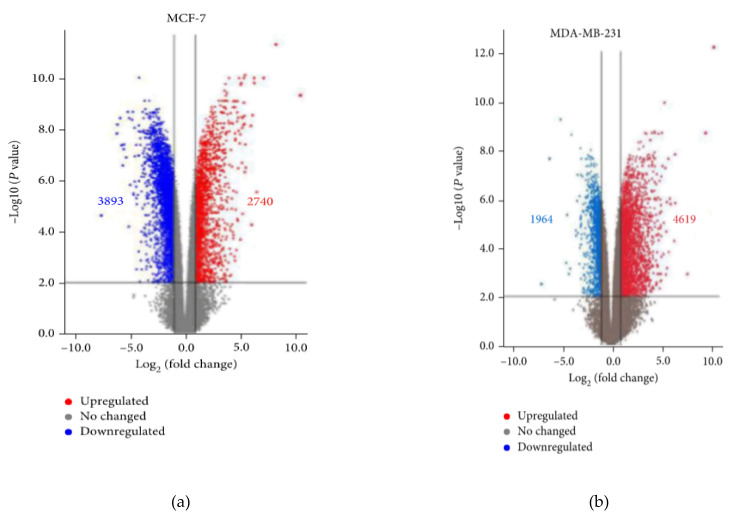
Differential expression hierarchical clustering analysis (**a**) Volcano plots of the transcription levels of genes in control and 40 μM curcumin-treated MCF-7 cells. (**b**) Volcano plots of the transcription levels of genes in control and 40 μM curcumin-treated MDA-MB-231 cells (Figure 14 is taken from the work R. Li [228]).

The effects of sequencing the transcriptome of various cell types treated with turmeric were considered [228]. The results show large-scale changes in gene expression levels. For example, treatment of breast carcinoma cells showed increased expression of 2,740 genes and decreased expression of 3,893 genes in the MCF-7 cell line; in the MDA-MB-231 cell line, the expression of 4,619 genes was increased and 1964 genes were decreased. Curcumin has been found to enhance the expression of various ferroptosis target genes associated with redox regulation, especially hemooxygenase-1 (Figure 14).

**Figure 15:**
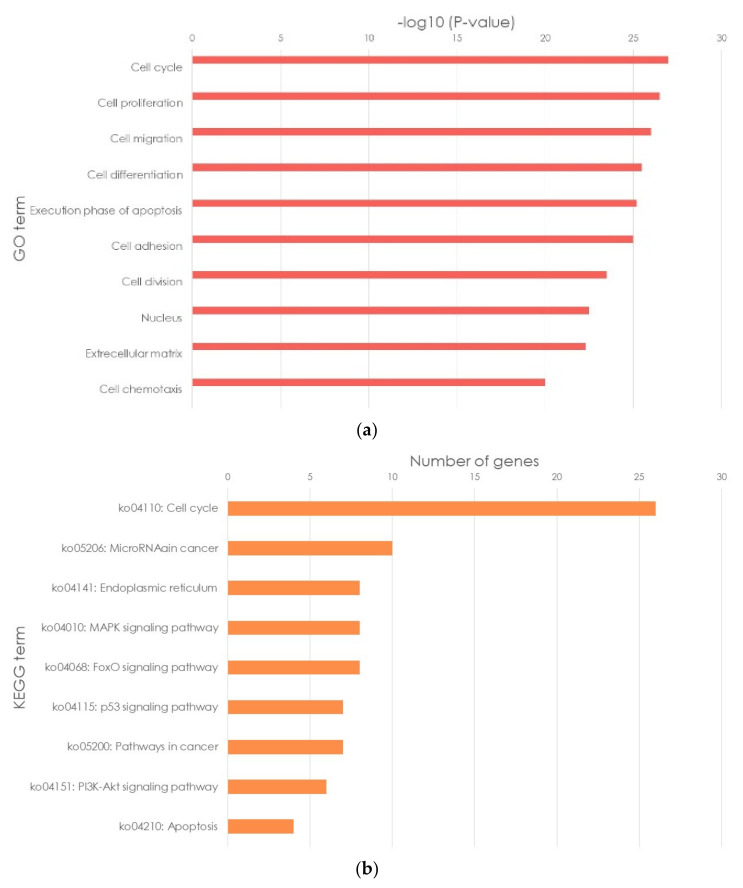
Bioinformatic analyses of the mechanism of curcumin in adrenocortical carcinoma: (**a**) The top ten enriched GO terms; (**b**) KEGG pathway analysis. (Figure 15 is taken from the work X. Huang [229]).

In [229], it was reflected that curcumin treatment of adrenocortical carcinoma cells revealed 385 differentially expressed genes: 114 genes with increased expression levels and 271 genes with decreased expression levels. The GO and KEGG pathway enrichment analysis showed that the predominant pathways associated with curcumin-induced apoptosis were “cell cycle,” “microRNA in cancer,” “MAPK signaling pathway,” and “endoplasmic reticulum (Figure 15).

Thus, the nutrient curcumin is able to modify the expression of genes controlling various signaling pathways in different types of cells and exert a variety of epigenetic effects.

**Figure 16:**
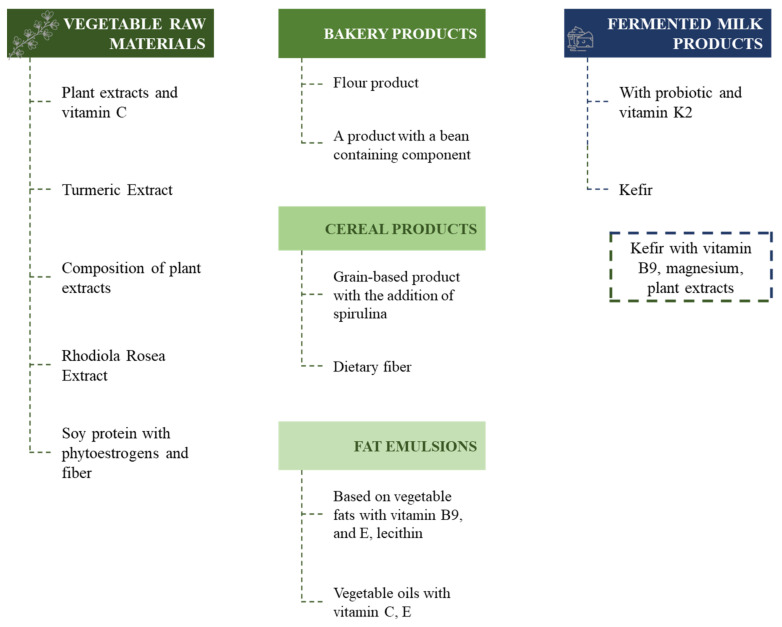
Functional products exhibiting cardioprotective activity found in patent research ([232,233,234,235,236,237,238,239,240,241,242,243,244,245,246,247,248,249,250]).

Since some of the abovementioned BAS are of animal or plant origin, there is a problem in the availability of a number of products to the consumer (geographical, climatic, economic problems). Consequently, it is promising to use functional products for the prevention of atherosclerosis and other metabolic diseases [230]. That is, foods that are not only a source of nutrients for the body, but also have potential health benefits [231]. Examples of the development of functional products exhibiting cardioprotective activity are shown in Figure 16.

The comparative analysis shows that functional products enriched with plant components (prebiotics), plant extracts and probiotics prevail. The prevailing compositions are based on vegetable raw materials added to both bakery and cereal products and fat emulsions. There are also fermented dairy products [251,252]. The mechanisms by which these products have a preventive and healing effect on the body are related to the content of nutrients (antioxidants, vitamins, etc.) and the elimination of food components that negatively affect health (fats, carbohydrates, etc.). The effect of consuming these products depends on the current state of health of the consumer, on the amount of nutrient in it and on the technology of manufacturing the product.

Functional products for the prevention of a number of diseases should undergo the following stages of evaluation, as reflected in [31]:

1. Assessment of composition (qualitative and quantitative analysis of macro- and micronutrients).
2. Evaluation of *in vitro* properties (antioxidant properties, antimicrobial properties, etc.).
3. Bioavailability and pharmacokinetic studies (absorption, distribution in tissues, etc.).
4. In vivo studies in animal models (pharmacokinetics, toxicology, intervention in disease models).
5. Human studies (observation of safety, bioavailability, cardioprotective potential, etc.)

To understand the mechanisms of the cardioprotective effects of BAS and their functional products, it is necessary to conduct experiments using different model objects under *in vitro* and *in vivo* conditions.

### 3.3. Model Organisms Applicable to Atherosclerosis Research

Animal models for studying the pathogenesis and prevention of atherosclerosis should have [253,254]:

1. Compatibility with human anatomy and physiology;
2. Relative ease of maintenance and affordable cost;
3. Lipid metabolism, genetic similarity, and a similar morphology of lesion development to that of humans;
4. Used in medical and pharmaceutical research to produce results that can be extrapolated to humans.

### 3.4. Animal Models to Study the Pathogenesis of Atherosclerosis

Table 4 reflects the rodent species used as models to study the relationship between atherosclerosis and nutrition.

Table 5 shows large animals used as models to study the relationship between atherosclerosis and nutrition.

Since the use of these models requires a lot of time, material and other costs, small mammals (mice, rats, rabbits), as well as large animals (pigs and non-human primates), despite all their advantages, are not suitable for the large-scale screening of potential cardioprotective substances. An alternative is to use smaller objects, such as danio fish (Table 6).

Several studies showed that danio fish can be used to study the development of atherosclerosis and methods for its dietary prevention and treatment. The obtained data reveal that, with an appropriate diet (with a high cholesterol content), cholesterol and macrophages accumulate in blood vessels with an increase in the expression of inflammatory factors’ mRNA (V TNF-A and IL-6) and vascular cell adhesion molecules (vcam-1b), followed by the thickening of the intima of blood vessels ad the formation of plaques [263]. With a high cholesterol diet, hypercholesterolemia, oxidation of lipoproteins and the formation of fat bands in adult danio fish were observed [264]. Genes involved in the metabolism of lipoproteins and lipids, such as *APOB*, *APOE*, *APOA1*, *LDLR*, *APOC2*, *LPL*, *LCAT* and *CETP*, are preserved in danio fish [265].

#### Experiments on Cell Models under *In Vitro*

It is possible to study the interaction of lipid and nutrient metabolism in cellular models. These models are convenient because research on them does not require ethics committee approval, they are relatively inexpensive and are easy to cultivate. So, in [269], the inevitability of the anti-atherosclerotic effect was obtained for the active occurrence of plant origin on a cellular model based on a natural culture of the human aorta. In [144], the atherosclerotic properties of baicalin *in vitro* on human monocyte cells THP-1 were studied. In [270] the ability of ethylpyruvate to inhibit LDL oxidation on human vascular endothelial cells, EA.hy926, was evaluated. In the work H.T., The effect of anthocyanins on the expression of the *PON1* gene was studied on a Huh7 liver hepatoma cell culture [271]. In [266] it has been shown that studies are often carried out on liver HepG2 cells, intestinal Caco2 cells, and 3LT3 adipocyte cells. However, cell lines consist of cells of the same type, and they cannot duplicate the cellular heterogeneity of the whole organ, that is, there are limitations in the metabolism of lipids for the investigated candidate nutrients that exhibit anti-atherosclerotic effects [266]. Therefore, for an accurate understanding of the pathogenesis of atherosclerosis and the mechanisms of the influence of nutrients, it is necessary to conduct studies both on cell models and on animal objects.

### 3.5. The Role of the Microbiota of the GIT in the Pathogenesis of Atherosclerosis

The microbiome of the GIT affects the body metabolism and its healthy state. So, SCFAs that regulate glycemic and lipid metabolism are involved in maintaining an intact intestinal barrier, maintaining intestinal pH, and regulating the immune system and inflammatory responses [7,17,176,272,273,274]. High levels of TMAO are known to increase the risk of atherogenesis. TMAO is formed from TMA, which is produced by the gut microbiota from choline, phosphatidylcholine, carnitine and betaine [275]. Therefore, the more of these substances in the diet, the higher the level of TMAO [276].

The microbiota continuously changes its qualitative and quantitative composition during human life under the influence of a number of factors. For this reason, certain food components change the composition of the microbiota, both positively and negatively. Consumption of animal protein reduces the amount of SCFAs [26]. Probiotics and prebiotics in food and FF that can modulate the composition of the microbiota are of considerable interest [14,277,278,279,280]. The probiotics that exhibit cardioprotective properties are presented in Table 7.

The cardioprotective effect of probiotics is due to the maintenance of an optimal number of beneficial representatives of the microbiota of the GIT (modulation of microbiota composition), regulation of microbial metabolites, reduction of anti-inflammatory cytokines, total cholesterol, LDL and TMAO levels, etc. [306]. The importance of the normal functioning of the gastrointestinal microbiota and its impact on the overall health of the body, in particular atherosclerosis, promotes the inclusion of probiotic, prebiotic components in a personalized diet that regulates its qualitative and quantitative composition and metabolites, but also limits the consumption of a number of substances (e.g., animal proteins).

### 3.6. Recommendations for the Prevention of Atherosclerosis

Prescribing an effective preventive, individualized diet requires not only the accurate interpretation of the results of nutrigenetic testing, but also the consideration of all health-related features of the body (presence of chronic diseases, blood chemistry, family history), as well as knowledge of generally accepted, universal anti-atherosclerotic, cardioprotective recommendations. The following general recommendations for the prevention of CVD, particularly atherosclerosis can be considered as a basis for personalized analysis [133]:

Recommendations of the Japan Atherosclerosis Society (JAS) [307,308];
Recommendations of the Russian Society of Cardiology [309];
Recommendations of the Brazilian Society of Cardiology [310];
Recommendations of the American College of Cardiology [311];
Recommendations of the Canadian Cardiovascular Society [312];
Recommendations of the European Atherosclerosis Society [313,314].

These guidelines describe the main risk factors for CVD. Methods of diagnosis, prophylaxis and treatment (with medications) are given, as well as a description of dietary recommendations. Dietary recommendations include limiting/eliminating the intake of salt, confectionery and other products containing trans fats; enriching the diet with polyunsaturated fatty acids; limiting the use of products with excessive carbohydrates (mono-, disaccharides); replacing animal protein with vegetable protein; systematic use of fruits and vegetables, etc. To create personal recommendations, it is necessary to study generally accepted recommendations, followed by their modernization, taking into account the individual characteristics of the consumer.

## 4. Conclusions

**Figure 17:**
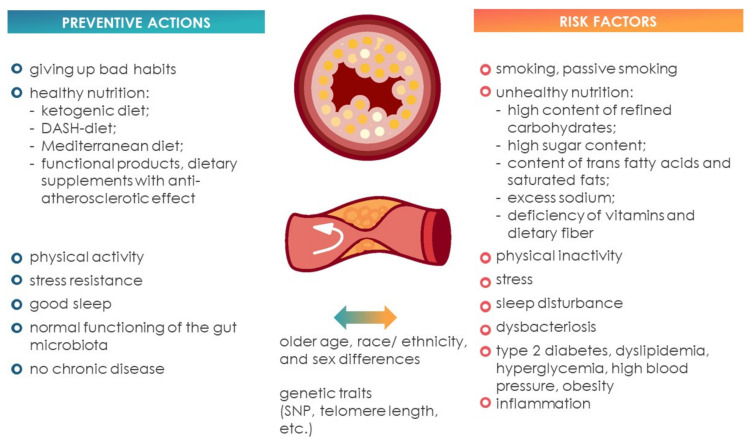
Factors influencing the development of atherosclerosis.

Nutrition affects a healthy body. The current level of scientific and technological development contributes to the formation of a personalized approach to nutrition for the prevention of metabolic diseases, including atherosclerosis [9]. Atherosclerosis is a multifactorial disease, as generalized in Figure 17, and the consideration of atherosclerosis development factors contributes to the formulation of preventive measures. A special role in the personalized approach is played by nutritional genetics, represented by three areas (nutrigenetics, nutrigenomics and epigenetics). Nutrigenomics determines the optimal diet from a range of alternative dietary patterns. Nutrigenetics and epigenetics provide important information about a consumer’s genetic makeup that helps experts select the best personalized diet for that individual to prevent atherosclerosis [27]. In the nutrigenetic study, the main tool is the analysis of SNP genes involved in the pathogenesis of atherosclerosis [32]. Such epigenetic factors as DNA methylation and mRNA targeting are also targets for the prevention of atherosclerosis.

The survey presents dietary models used to prevent CVD: the KD, DASH-diet and the MD (which is particularly popular). Other diets are also possibly suitable for preventing CVD: the Japanese diet, Scandinavian diet, portfolio diet, vegetarian diet, diet with LGI foods. The review found no or minimal reliable data on the clinical significance of these diets. For many consumers, the choice of an optimal diet, namely, the use of a number of products, is difficult because of geographic, climatic and financial constraints. Consequently, the development of functional products that exhibit cardioprotective and anti-atherosclerotic potential is relevant. In the course of the work, it was found that plants rich in cardioprotective BAS (baicalin, resveratrol, curcumin, quercetin, chlorogenic acid) can be used as raw materials. The animal raw materials are also a source of these substances (protein hydrolysates, squalene, coenzyme Q10, omega-3).

Mostly, functional products for the prevention of atherosclerosis belong to the group of dairy, bakery and cereal products. To give them bioactivity in their composition, researchers have introduced plant extracts, prebiotics, and probiotics. Research is needed to study the anti-atherosclerotic potential of these BAS and functional products.

This work considered model organisms used to study the pathogenesis of atherosclerosis. The choice of the model (small, large animals, cellular models) depends on the nature and funding of the study to be performed. Of course, the best extrapolation of human research results is achieved with pigs and non-human primates. However, the application of these models has serious limitations in ethics, cost and duration of research. The use of small animals (mice, rats, rabbits) is deprived of some of these drawbacks, but microscopic examination of atherosclerotic lesions can only be performed post mortem. In addition, neither small nor large animals are suitable for the large-scale screening of potential anti-atherosclerotic drugs. An alternative model is the danio fish, which have high fecundity, a transparent body at the larval stage and genetic similarity to the human genome [268]. Selecting a model for the study should be based on the characteristics of each model, its ability to reproduce the disease under study, the available budget, and time [259]. For a complete review of the pathogenesis and prevention of atherosclerosis, it is necessary to conduct experiments first on cellular models, then on animal objects.

Gastrointestinal microbiota plays an important role in maintaining a healthy body. In the course of the work, probiotic and prebiotic strains were isolated, modulating the qualitative and quantitative composition of microbiota, affecting intestinal metabolites (TMAO, etc.), reducing levels of anti-inflammatory cytokines, total cholesterol and LDL. These prebiotics included mannans, α-cyclodextrin, glucosides, etc. Probiotics include lactic bacteria of the genera *Akkermansia*, *Lactobacillus*, *Bifidobacterium*, *Enterococcus*, *Pediococcus.* Therefore, for the formation of personalized nutrition, it is necessary to include FF in the diet that can normalize the work of the microbiota of the GIT.

During the work, restrictions were highlighted that inhibit the personalization of nutrition as a preventive means.

**Restrictions related to genetic characteristics**: Theoretically, there are a large number of factors (genetic, epigenetic, etc.) related to nutrition that leads to the development of atherosclerosis. As a result, there are difficulties with the evidence base. Difficulties are due to the fact that one and the same gene, and one and the same mutation influences a large number of processes occurring in the body. Also, difficulties due to preclinical and clinical studies, low reproducibility, high cost, etc., should be mentioned [315]. In addition, the optimal number of genetic markers has not been established, the study of which contributes to the formation of personal nutrition.

**Restrictions related to nutrigenomics**: There is a large number of BAS with a possible cardioprotective effect. It is difficult and costly to test precisely their anti-atherosclerotic potential. The work needs model organisms (cells, small, large animals) on which large-scale studies of these substances are possible. These models’ availability is related to a number of ethical and resource costs, with difficulties in extrapolating data to humans. More randomized, controlled long-term studies are necessary to determine the true effect of macronutrient foods on atherosclerosis risk. In addition, it is difficult to assess the individual impact of a particular BAS, since the human or animal body is affected by a large number of factors related to diet and other characteristics.

**Restrictions related to dietary factors:** The modern product market contains high-calorie products with excessive salt content, trans fats, products that do not contain vitamins, macro- and microelements. The products traditionally included in a number of diets (DASH-diets, MD, Scandinavian and other diets) are most often not available to large segments of the population. The reasons for inaccessibility are financial, climatic or geographical restrictions. Difficulties are evident with conducting clinical trials of diets.

**Restrictions related to resource limitations:** Specialists competent in various fields or a collaboration of specialists of various profiles are needed to form a personalized approach to nutrition for the prevention of atherosclerosis. Specialists are needed who are able to correctly interpret the results of nutrigenetic testing.

Despite the presented restrictions, work in the field of personalized nutrition for the prevention of atherosclerosis is being actively implemented, since personalized nutrition is a promising preventive measure. It should be pointed out that not only is nutrition necessary for the prevention of metabolic diseases, but is important to adhere to other indicators of a healthy lifestyle, observe circadian rhythms, give up bad habits and load the body with physical activity [9,23,316,317].

As to future prospects of this work, the following research directions seem to be topical. In Kemerovo region, a large coal mining basin, more than 70,000 miners are working and living, and their specific dangerous working conditions may generate professional respiratory diseases. Also, the working conditions may be an additional risk factor for the appearance of multifactorial diseases, including atherosclerosis. Accordingly, the possible mechanisms of atherosclerosis formation, dependent on working conditions and nutrition, should be considered for this specific population group. The search of biologically active substances (BAS) for the prevention of atherosclerosis is promising in numerous biological resources of Siberia. Optimal concentrations of BAS for the prevention of different diseases should be defined for their incorporation to the diets. What is particularly promising is the research direction aimed at the design of markers indicating early stages of atherosclerosis that can be helpful in its diagnostics and treatment.

## Figures and Tables

**Table 1:** List of plants and their metabolites showing cardioprotective activity.

Substance	Model Object	Function	Resource
Baikalin	male rabbits	Reducing the size of atherosclerotic lesions and lipid accumulation in the carotid arteries. Baicalin increased the expression of *PPARγ* and *LXRα*, *ABCA1* and *ABCG1.*	[144,145]
	Human monocyte cells THP-1	Effect on intracellular lipid accumulation	
Resveratrol	Human monocyte cells THP-1	Suppression of gene expression *LPL* and *SR-AII* in macrophages, upregulation of genes *ABCG1* and *ABCA1*	[146,147,148,149,150,151,152,153]
	Male ApoE-/- mice	Stimulation of the thickening of the coronary artery wall and decreased the areas of atherosclerotic lesion on aortas. Resveratrol decreased the number of CD4+ T cells in peripheral blood, decreased the expression of *CD25* and *CD44*. *In vitro*, resveratrol decreased the expression of *Ki67*, *CD25* and *CD44* in CD4+ T cells. Resveratrol increased the secretion of *IL-2*, *IL-10* and *TGF-β1*, decreased *IL-6*. Resveratrol decreased both the mRNA and protein level of Dnmt1 and Dnmt3b in CD4+ T cells.	[154]
Grape seed extracts	streptozotocin-induced diabetic rats	*PON1* activities	[155]
Red wine polyphenolic compounds	hyperhomocysteinemic mice	reduced plasma homocysteine levels, *PON1* activities	[156]
Curcumin	PON1-Huh7 cells, female B6C3F1 mice	A two-week diet with curcumin did not increase mRNA and *PON1* protein levels in the liver. Curcumin potent *PON1* inducer in cultured cells *in vitro*	[157]
Liposomes encapsulating atorvastatin calcium and curcumin	Human aortic endothelial cells, ApoE-/- mice	Decreased the areas of atherosclerotic lesion on aortas. Suppression of adhesion molecules (E-selectin and *ICAM1*) and plasma lipid levels. Reduction of foam cell formation and the secretion of inflammatory factors (*IL-6* and *MCP-1*) by blocking monocyte migration into the intima.	[158]
Quercetin	*ApoE3* and *ApoE4* transgenic mice	Hepatic mRNA and protein levels of *PON1* were significantly lower in apoE4 as compared to *APOE3* mice. Feeding quercetin-enriched diets induced hepatic *PON1* gene expression with a tendency for greater induction in *APOE3* as compared to *APOE4* mice.	[159]
	Macrophages RAW264.7	The expression of LC3-II/I and BECLIN1 were increased, which was consistent with the ability of quercetin to promote autophagy. Quercetin can inhibit the formation of foam cells induced by ox-LDL.	[160]
	ApoE-/- mice	Quercetin prevents the development of atherosclerosis in *APOE*-/- mice by regulating the expression of *PCSK9*, *CD36*, *PPAR*γ, *LXRα* and *ABCA1*.	[161]
	ApoE-/- mice, C57BL/6J mice	Reduced the levels of total cholesterol, TG, LDL.	[162]
Onion extract (*Allium cepa* L.)	male Wistar rats	*PON1* activities	[163]
Garlic extracts (*Allium sativum*)	mouse macrophage cell line RAW264.7	Strong antioxidant effects: high ABTS and DPPH radical scavenging activities. Inhibition of *COX-2* and *5-LOX* activities.	[164]
	Male ApoE-/- mice	Decreased the areas of atherosclerotic lesion on aortas. Reduced the levels of total cholesterol, TG.	[165]
*Salvia miltiorrhiza*	ApoE-/- mice	Decreased the areas of atherosclerotic lesions on aortas.	[166]
Extracts from *Astragalus membranaceus*	Male sprague-dawley rats	Extracts improved cardiac function, attenuated the oxidative injury via a decrease in MDA, a maintenance in SOD, and a reduction in free radical-induced myocardial cell injury.	[167]
Bilberry extract	ApoE-/- mice	Reduction hypercholesterolemia. Bilberry extract supplementation affected the expression of genes involved in oxidative stress, inflammation or cell adhesion/migration.	[168]
Berries (bilberries, black currant or strawberry, lingonberries, chokeberry and raspberry)	human	Berry consumption inhibited platelet function. Increased HDL-C concentrations. Decreased Systolic BP.	[169]
Lingonberries	ApoE-/- mice	Decreased triglyceride levels and amount of atherosclerotic plaques decreased. Increased expression of *CYP7A1*. Lingonberries increased the cecal relative abundance of bacterial genera *Bacteroides*, *Parabacteroides* and *Clostridium*. Decreased the cecal levels of total SCFAs.	[170]
Black rice pigment fraction	ApoE-/- mice	Decreased the areas of atherosclerotic lesion on aortas. Reduced the levels of total cholesterol. Increased HDL-C concentrations.	[171]
Rice protein isolate	ApoE-/- mice	Increased expression of antioxidant enzymes: superoxide dismutases, glutathione peroxidases, glutathione reductases.	[172]
Mulberry leaf-related extracts	isolated human peripheral blood lymphocytes, cell cultures (HAECs)	Strong antioxidant activity: downregulated intracellular redox-dependent signaling pathways in HAECs upon *TNF-α* stimulation.	[173]
Delphinidin	Human monocyte cells THP-1	Pre-treatment with delphinidin decreased the ox-LDL-induced up-regulation of the expression of ICAM1 and P-selectin, and the enhanced adhesion and transmigration of monocytes.	[174]
Inulin	Male *ApoE*-/- mice	Decreased the areas of atherosclerotic lesion on aortas. Reduced the levels of total cholesterol, TG	[175]
	People with chronic kidney disease	Reduced the levels in serum insulin, fasting glucose levels. total serum cholesterol, TG, homocysteine, C-reactive protein, higher HDL	[176]
	male golden syrian hamsters	Reduced the levels of total cholesterol, TG. Inulin caused distinctive changes in the circulating bile acid profiles and modestly enhanced fecal bile acid excretion.	[177]
	human	Reduced the levels of plasma triacylglycerol concentrations and hepatic lipogenesis.	[178]
Oligofructose-enriched inulin	females with type 2 diabetes	Increase in total antioxidant capacity. Significant decrease in fasting plasma glucose, HbA1c, total cholesterol, LDL-C, total cholesterol/HDL-C ratio, LDL-C/HDL-C ratio, malondialdehyd.	[179]
Oligofructose	male Wistar rats	Oligofructose improved gastrointestinal structure and function. Oligofructose attenuated H-induced increases in inflammatory cell infiltration in the heart and liver, lipid droplets in the liver and plasma lipids as well as impaired glucose and insulin tolerance. Reduced fasting blood glucose concentrations, systolic BP.	[180]
Mannan	Female *E3L.CETP* mice	Mannan decreased the onset of atherosclerosis development via lowering of plasma cholesterol levels. Mannan increased the abundance of cecal bacteroides ovatus and butyrate.	[181]
Vitamin A	*ApoE*-/- mice	Vitamin A-deficient diet significantly increased both plasma cholesterol concentrations and the atherosclerotic lesion area at the aortic sinus. Dietary vitamin A fortification inhibited the elevation in plasma cholesterol and retarded atherogenesis in mice fed the vitamin A-deficient diet.	[182]
Lycopene and astaxanthin	healthy male sprague-dawley rats	Lycopene and astaxanthin reduced total cholesterol, LDL-C, and TG and increased HDL-C level significantly.	[183]
Vitamin C	*Gulo(-/-)Apoe(-/-)* mice	Chronic vitamin C deficiency does not influence the initiation or progression of atherosclerotic plaques but severely compromises collagen deposition and induces a type of plaque morphology that is potentially vulnerable to rupture.	[184]
Niacin	Male *ApoE*-/- mice	Niacin inhibits vascular inflammation and apoptosis of VSMCs via inhibiting the NF-κB signaling and the FAK signaling pathway, respectively, thus protecting *ApoE*-/- mice against atherosclerosis.	[185]
Serum folate and vitamin B12	hypertensive people	The risk of first ischemic stroke was significantly higher in hypertensive patients with low levels of both folate and B12.	[186]
9-cis-β-carotene rich alga *Dunaliella*	*ApoE*-/- mice	Reduced the levels of total cholesterol. Decreased the areas of atherosclerotic lesion on aortas.	[187]
Lupin	*ApoE*-/- mice	Lupin protein reduce the calcification of atherosclerotic lesions in *APOE*-deficient mice.	[188]
Ginger extract	*ApoE*-/- mice	Reduced the levels of total cholesterol, TG, LDL. Decreased the areas of atherosclerotic lesion on aortas.	[189]
Chlorogenic acid	*ApoE*-/- mice, RAW264.7 cells	Treatment with chlorogenic acid reduced the area of atherosclerotic lesions and vascular dilatation in the aortic root; reduced plasma levels of total cholesterol, TG, and LDL-C as well as inflammatory markers. Treatment with chlorogenic acid stimulated cholesterol efflux from RAW264.7 cells. Treatment with chlorogenic acid significantly increased the mRNA levels of *PPARγ*, *LXRα*, *ABCA1* and *ABCG1* as well as the transcriptional activity of *PPARγ.*	[190]
Perilla Oil (*Perilla frutescens*)	*ApoE*-/- mice	Reduced the levels of total cholesterol, TG, LDL. Observed reduced fatty streak lesion size at the aortic sinus; enhancement expression *eNOS*; reduction expression *iNOS*, *ICAM1*, *VCAM1*.	[191]
Ethanolic extract of propolis	*ApoE*-/- mice	Reduced the levels of total cholesterol, TG. Enhancement expression *eNOS*, *IL-17*, reduction expression *IL-6*	[192]
2,3,5,4′-Tetrahydroxy-stilbene-2-O-β-D-glucoside (*Polygoni Multiflori Radix)*	*ApoE*-/- mice	Suppression of formation of an atheromatous plaque. Reduction of expression of IL-6, TNF-α, VCAM1, MCP-1 in serum and CCRA expression in aortic tissue. Regulation of the composition of the overall gut microbiota, such as *Firmicutes*, *Bacteroidetes*, *Tenericutes, Proteobacteria phyla*, *Akkermensia genera* and *Helicobacter pylori*.	[193]
Berberine	*ApoE*-/- mice	Decreased the areas of atherosclerotic lesion on aortas. Reduced the levels of total cholesterol, LDL-C levels. Decreased the pro-inflammatory cytokines tumor necrosis factor-alpha, interleukin (IL)-1β, IL-6 and increased anti-inflammatory IL-10 and adiponectin levels. Modification of the community compositional structure of the gut microbiota: elevated potential for lipid and glycan metabolism and synthesis of SCFAs and reduced potential of TMAO production.	[194]
	macrophage THP-1	Promotes the outflow of cholesterol by increasing the formation of reactive oxygen species, which subsequently causes autophagy through the PI3K/AKT/mTOR signaling pathway both in “normal” macrophages and in macrophages loaded with lipids (foam cells)	[195]

**Table 2:** BAS—cardioprotectors of non-plant origin.

Substance	Model object	Function	Resource
salmon protein hydrolysate	Female *ApoE*-/- mice	Decreased the areas of atherosclerotic lesion on aortas. Reduced mRNA level of *ICAM1* in the aortic arch. Decreased the plasma concentration of IL-1β, IL-6, TNF-α and GM-CSF.	[196]
hen eggs enriched naturally with conjugated linoleic acid	*ApoE*-/-, *LDLR*-/- mice	Reduced the levels of total cholesterol. Reduced number of atherogenic macrophages and increased the area occupied by smooth muscle cells in atherosclerotic lesions	[197]
Anchovy (*Engraulis encrasicolus*) Protein Hydrolysates	*ApoE*-/- mice	Reduced the levels of total cholesterol and reduction in plaque size.	[198]
Chicken protein hydrolysate	*ApoE*-/- mice	Decreased the areas of atherosclerotic lesions on aortas	[199]
Squalene	*ApoE*-/- mice	Decreased the areas of atherosclerotic lesion son aortas in males, but not in females. Reduced the levels of total cholesterol, TG regardless of gender.	[200]
Coenzyme Q10	*ApoE*-/- mice	Reduced the levels of total cholesterol, TG, LDL-c	[201]
omega-3 PUFAs	male	Reduced the levels of TG and total cholesterol levels, decreased IL-6 levels, increased PAI-1 levels	[202]

**Table 3:** BAS that are epigenetic modifiers.

Substance	Action	Model Organism	Diet	Effect	Resource
Resveratrol	activator SIRT1	E3L mice *CETP*	diet with resveratrol (0,01% *w/w*)	Decreased the areas of atherosclerotic lesions on aortas. Increased plaque stability. Reduced the levels of total cholesterol levels.	[203]
		Fbn1(C1039G/+) MFS mouse model	resveratrol (0,1 mg/)mL was injected into drinking water	Reduction of aortic elastin ruptures and reduction of microRNA-29b expression. Reduction of aortic aging. Increased activation of *SIRT1* gene (sirutin 1, the expression of which increases insulin sensitivity)	[204]
	The resveratrol effect was surprisingly mediated by the aryl hydrocarbon receptor (AhR) and an unconventional AhR responsive element in the PON-1 gene promoter.	hepatoma cell line HuH7	HuH 7 cells were treated with 10 mol/l of resveratrol (0.1% ethanol) for 48 h in a conventional culture medium	An increase in the level of *PON-1* mRNA, a significant increase in the activity of the promoter of the *PON1* gene, in the expression of the *PON1* gene.	[205]
Quercetin	ингибитop DNMT	wild-type (WT) C57BL/6 mice, *ApoE-/-* mice	high-fat diet supplemented with (0.05% *w/w*) quercetin	Reduced oxidative stress, increased endothelial *eNOS* activity, and increased heme oxygenase-1 protein expression in aortic tissue	[206]
Vitamin C	activator TET2	rabbits	feeding 100 mg of cholesterol per day and the introduction of various doses of ascorbic acid (0.5 and 15 mg/100 g of body weight)	Decreased atherogenicity: reduced accumulation of lipids	[207]
		human hepatoma cell line HepG2	treatment of cell cultures with vitamin C (0, 400, 800 μM) for 24 h	Vitamin C regulates (*LPA*) synthesis, down-regulated *APOA* expression, induces global DNA and EIK1 promoters of hydroxymethylation in HepG2 cells.	[208]
Vitamin C, Vitamin E	-	*ApoE*-/- mice	vitamin C (120 mg/kg per day) and vitamin E (210 mg/kg per day) were introduced into drinking water	Reduced expression of *VEGF*, which affects the rate and extent of atherosclerotic plaque formation	[209]
Curcumin	Broad spectrum epigenetic modulator	*LDLR-/-* mice	high-fat, high-cholesterol Western diet with oral curcumin (100 mg/kg in 0.5% carboxymethylcellulose)	Decrease in the level of lipopolysaccharides in the blood. Improved intestinal barrier function. Reducing the size of atherosclerotic plaques	[210]
		*ApoE*-/- mice	high-fat diet supplemented with curcumin (0.1% by weight)	Decreased the areas of atherosclerotic lesion on aortas Decreased expression of *TLR4*, NF-kB, *IL-1β*, *TNFa*, *VCAM1*, *ICAM1*.	[211]
		hypertension rat model	intraperitoneal injection (200)µL every 2 days for 56 days at concentrations of 25, 50, 100, 200 and 400 mg/kg body weight	decreased expression of MMP-2, HDAC1 genes and TGFβ	[212]
		Male *ApoE*-/- mice, human monocytic THP-1 cells	received daily curcumin (20 mg/kg body weight) by gastric gavages for 16 weeks together with high-fat diet	regulates foam cell autophagy, inhibits inflammation and lipid content	[213]
Pomegranate juice	reducing oxidative stress: inhibition of LDL oxidation, preservation of paraoxonase activity	patients with asymptomatic severe carotid artery stenosis	Taking pomegranate juice for 1 year. Taking pomegranate juice for 3 years.	Reduction in oxidative stress was demonstrated already after 1 month taking pomegranate juice. Increase in serum *PON1* activity (up to 91% after 3 years taking pomegranate juice). Reduction in the patients systolic BP.	[214]
		male	pomegranate juice consumption for ≤2 and 14 wk	decreased LDL susceptibility to aggregation and retention and increased the activity of serum paraoxonase by 20%	[215]
		*ApoE*-/- mice		Reduced cellular lipid peroxidation and superoxide release. The uptake of oxidized LDL and native LDL by mouse peritoneal macrophages obtained after pomegranate juice administration was reduced by 20%. Finally, pomegranate juice supplementation of E0 mice reduced the size of their atherosclerotic lesions by 44% and also the number of foam cells compared with control E0 mice supplemented with water.	
TMAO	TMAO is associated with risk of cardiovascular disease	Cell lines (HepG2 and THP-1), Peripheral blood mononuclear cells	addition of TMAO at various concentrations	The TMAO modulates the expression of miRNAs related to lipid metabolism, atherosclerosis, and CVD	[216]

**Table 4:** Small mammals used as models to study the relationship between atherosclerosis and nutrition.

Model Object	Advantages		Disadvantages		Resource
	General	Individual	General	Individual	
**Mice**	Low cost, simple in-house maintenance. Short gestation period. Large numbers of progeny. Large proportion of genes is in homology to humans. Ease of genetic manipulation and its ability to monitor atherogenesis in a reasonable time frame, Similar morpology of lesion development.	most rapid reproduction	Do not express CETP. Show only minor plaque development in the coronary and carotid arteries. Absence of intra-plaque neovascularization and hemorrhage. Different atherosclerosis generation times (for example, months in genetically modified mice and years in humans	The small size of mice can be limiting for some practical investigation procedures. Difference in the morphology of the arterial tissue due to the insignificant size of the vessels. Relatively resistant to the development of atherosclerosis. Substantial genetic differences in the lipid metabolism between mice and man. Most of the cholesterol is transported in HDL-like particles.	[254,255,256,257,258]
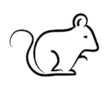					
Kpыcы		Relatively suitable size—invasive procedures and sample collection are easier to perform in rats compared with small-size mice		No high cholesterol production. Frothy cells with more fatty streaks and more macrophages	[253,259]
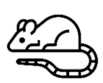					
Kpoлики		Average body size: large artery allows for clinical evaluation. Rabbits are phylogenetically closer to humans than rats and mice.		Relative deficiency of hepatic lipase. Massive inflammation and hepatic toxicity that develop in response to long-term cholesterol-rich feeding aimed to induce hypercholesterolemia. Different cardiovascular physiology to humans: HDL as the predominant plasma lipoprotein, absence of Apo AII, low hepatic lipase activity. Plaque lesion dissimilar to humans: foam cells with a more fatty streak and macrophage-rich, advanced lesions (e.g., fibrosis and hemorrhage and ulceration) are not seen. Not always responsive to dietary cholesterol.	[254,255,256,258,260]
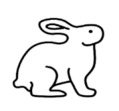					

**Table 5:** Large animals used as models to study the relationship between atherosclerosis and nutrition.

Model Object	Advantages		Disadvantages		Resource
	General	Individual	General	Individual	
Porcine	Similar heart size and cardiovascular anatomy. Spontaneously develop atherosclerosis with an accelrated rate when fed with atherogenic diet. Omnivorous diet. Easier to carry out imaging, e.g., Ultrasound, CT and MRI compared to smaller species. Similar hemodynamics and pathogenesis to humans: lesion location, morphology and content. Highly defined genotypes for genetic manipulation	Highly defined genotypes for genetic manipulation	Significant ethical concern. Relatively expensive and more difficult to handle. Large in size, which limits their practical use. Long lifespan and hence a long period of time needed for induction of atherosclerosis.	*Apo II* deficiency. Plaque development mostly ends in the foam cell stage. Thrombosis due to plaque rupture is rare. Toxic diet needed for induction of atherosclerosis.	[255,257,258,260]
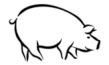					
Non-human primates		Closest phylogenetic relationship with human. Highest resemblance to human atherosclerotic clinical condition. Very similar plaque formation as compared to humans. Plaque formation in the coronary arteries.		Low availability.	
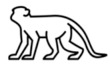					

**Table 6:** Advantages and disadvantages of danio fish used as a model organism for atherosclerosis pathogenesis.

Model Object	Advantages	Disadvantages	Resource
Zebrafish (*Danio rerio*)	They are small and thin enough to be imaged with a confocal microscope at high resolution. Zebrafish larvae are optically transparent until about 30 days postfertilization, which enables observation of disease progression *in vivo*. A large number of offspring, extrauterine fertilization and rapid development. Maintenance is relatively inexpensive and large numbers of animals can be produced for each experiment. Ease of genetic manipulation. Expresses genes relevant to lipid metabolism (MTTP, CETP et al.). Lipid metabolism in general, is surprisingly similar to that in humans. Lipids, rather than carbohydrates, are mainly used as an energy source.	Blood Samples can only be collected in small numbers from zebrafish older than 45 days. Lack of nutritional control. Dietary disorders in zebrafish are difficult to translate into human nutritional recommendations due to underlying factors in significant macro and micronutrients. Lipid accumulation in the postnatal stage can occur differently in zebrafish and humans. The zebrafish leptin protein is only 19% identical to the human protein and is not expressed in zebrafish adipose tissue, neither does its receptor. Zebrafish also produces only apoB-100, but not apoB-48, thus its chylomicrons are coated with apoB-100 which alters clearance in liver. Moreover, there are 2 apoB paralogues in the genome. Zebrafish is a poikilothermic organism, so lipid metabolic pathways cannot be discussed in the thermoregulation context, since temperature also affects growth, metabolic rate, and body fat composition in this poikilothermic animal.	[261,262,263,264,265,266,267,268]
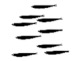			

**Table 7:** List of probiotics consisting of a number of strains that normalize the work of the gastrointestinal microflora and are used as an anti-atherosclerotic component.

Microorganism	Function	Form	Model object	Resource
*Akkermansia muciniphila*	Reduces inflammatory reactions, inhibits the proliferation and migration of macrophages, lowers blood cholesterol levels	Probiotic biomass	*ApoE*-/- mice	[281]
	Reduces body weight, reduction of total cholesterol and triglyceride levels, increased total B-cell population	Microbial suspension	*CETP*-/- mice E3L	[282]
*L. acidophilus*	Reduced the levels of LDL-c	Synbiotic capsule containing probiotic and inulin	Human	[283]
	Reduced the levels of total cholesterol	Microbiological inoculant	*ApoE*-/- mice	[284]
*L. plantarum*	Decreased intestinal–hepatic circulation of bile acid salts, reducing the bioavailability of cholesterol from the diet, reduced the levels of total cholesterol, inhibition of atherosclerotic plaque formation	Food additive	Human	[285]
	Reduced the levels of LDL-C	Probiotic in a capsule	Human	[286]
	Reduced the levels of total cholesterol	Probiotic suspension	Rats	[287]
	Improvement of vascular endothelial function, Reduction of systemic inflammation, reduced the levels of total cholesterol, LDL-C	The probiotic was part of a dietary supplement GoodBelly StraightShot (NextFoods, Inc., USA)	Human	[288]
*L. rhamnosus*, *L. plantarum*	Reduced the levels of LDL-C	Probiotic suspension	Human Caco-2 cells	[289]
*L.rhamnosus*	Reduced the size of atherosclerotic plaques, reduced the levels of total cholesterol.	Lyophilized probiotic powder	Mice	[290]
	Reduced the proinflammatory cytokine IL-1β concentration and cholesterol levels	Probiotic in a capsule	Human	[291]
*L. reuteri*	Reduced the levels of LDL-C	Probiotic in a capsule	Human	[292]
*L. fermentum*	Positive effect on the host immune system Changes in the content of reactive oxygen species in blood vessels, reduction of triglyceride levels in blood	Probiotic suspension	Rats	[293]
	Reduced the levels of total cholesterol, TG	Skimmed milk fermented with probiotic	Rats	[294]
*L. gasseri*	Reduced the levels of total cholesterol, the level of proinflammatory cytokine *IL-8*	Probiotic drug	Caco-2 and HT-29 cell lines	[295]
*B. animalis subsp. lactis*	Reduced the level of TMAO	Probiotic drug	Mice	[296]
	Reduced the level of TMAO	Nutritional supplement containing skim milk, glucose, inulin, dextrin, and silica in addition to probiotic	Human	[297]
*E. faecium*	Prevented a decrease in HDL levels	Probiotic suspension	Rabbits	[298]
*E. faecium*, *L. paracasei*	Reduced the levels of total cholesterol, TG, LDL-C, enhancement expression *CYP8B1*, *CYP7A1*, *SREBP-1*, SCD1 и *LDLR.*	Probiotic biomass	Rats	[299]
*P. acidilactici*	Regulates lipid metabolism, inflammatory processes	Probiotic suspension	Rats	[300]
*L. plantarum*, *S. thermophilus*	Reduced the levels of total cholesterol	Fermented soy milk with probiotics	Endothelial cells of the human umbilical vein	[301]
*L. acidophilus*, *L. casei*, *B.bifidum*	Reduced plasma glucose levels, serum insulin levels, change in HDL levels	Probiotic and Inulin	Human	[302]
		Probiotic in a capsule	Human	[303]
*L. gasseri*, *L. plantarum*, *L. helveticus*	Anti-inflammatory, immunomodulatory, hypocholesterolemic activity	Probiotic drug	Caco-2 and HT-29 cell lines	[304]
*L. brevis*, *L. kefianofaciens*, *L. helveticus*, *L. casei*, *L. plantarum*, *L. kefiri*, *Lactococcus lactic*, *S.unisporus*, *I. orientalis*	Reduction in the expression of *CRP*, *VCAM1*, *ICAM1*, modulation of lipid metabolism	Probiotic biomass	Rabbits	[305]

## Abbreviations

5-LOX	ALOX5 (arachidonate 5-lipoxygenase) is encoded by the ALOX5 gene
ABCA1	ATP-binding cassette sub-family A member 1 is encoded by the ABCA1 gene
ABCG1	ATP-binding cassette sub-family G member 1 is encoded by the ABCG1 gene
ABTS	2,2’-azino-bis(3-ethylbenzothiazoline-6-sulfonic acid)
ADD1	adducin 1 is encoded by the ADD1 gene
AKT	Protein kinase B (PKB)
ALDH2	Aldehyde dehydrogenase 2 is encoded by the ALDH2 gene
ANXA1	annexin A1 is encoded by the ANXA1 gene
APOA/LPA	lipoprotein A is encoded by the APOA gene
APOA1	Apolipoprotein AI is encoded by the APOA1 gene
APOB	apolipoprotein B is encoded by the APOB gene
APOC2	Apolipoprotein C-II is encoded by the APOC2 gene
APOE	apolipoprotein E is encoded by the APOE gene
BAA	Biologically active food additives
BAS	biologically active substances
BECLIN1	protein that in humans is encoded by the BECN1 gene
BMI	Body Mass Index
BP	blood pressure
CCR2	C-C chemokine receptor type 2
CCR5	C-C chemokine receptor type 5
CCRA	C–C chemokine receptor type 2 (CCR2) is encoded by the CCRA gene
CD25	Interleukin-2 receptor alpha chain (IL2RA) is encoded by the IL2RA gene
CD36	cluster of differentiation 36 is encoded by the CD36 gene
CD4+ T cells	T helper cells (Th cells)
CD44	cell-surface glycoprotein is encoded by the CD44 gene
CETP	Cholesteryl ester transfer protein is encoded by the CETP gene
COX-2	cyclooxygenase-2 (cytochrome c oxidase subunit II) is encoded by the COX-2 gene
CRP	C-reactive protein is encoded by the CRP gene
CT	computed tomography
CVD	cardiovascular diseases
CX3CR1	CX3C chemokine receptor 1 is encoded by the CX3CR1 gene
CXCL12	C-X-C motif chemokine 12 is encoded by the CXCL12 gene
CYP11B2	Aldosterone synthase is encoded by the CYP11B2 gene
CYP1A2	Cytochrome P450 1A2 is encoded by the CYP1A2 gene
CYP7A1	cytochrome P450 family 7 subfamily A member 1 is encoded by the CYP7A1 gene
CYP8B1	cytochrome P450 family 8 subfamily B member 1 is encoded by the CYP8B1 gene
CAT	Catalase is encoded by the CAT gene
DASH	dietary approaches to stop hypertension
DNA	Deoxyribonucleic acid
DNMTs	DNA methyltransferases
DPPH	2,2-diphenyl-1-picrylhydrazyl
DRD2	Dopamine receptor D2 is encoded by the DRD2 gene
DUSP1	Dual specificity protein phosphatase 1 is encoded by the DUSP1 gene
ECs	tndothelial cells
EC-SOD	Extracellular superoxide dismutase is encoded by the SOD gene
EIF2AK3	Eukaryotic Translation Initiation Factor 2 Alpha Kinase 3 is encoded by the EIF2AK3 gene
EIK1	ETS transcription factor ELK1 is encoded by the EIK1 gene
eNOS	Endothelial nitric oxide synthase
ERα	Estrogen receptor alpha
ERβ	Estrogen receptor beta
F2RL1	coagulation factor II (thrombin) receptor-like 1 is encoded by the F2RL1 gene
FADS2	Fatty acid desaturase 2 is encoded by the FADS2 gene
FAK	focal adhesion kinase
FF	functional foods
FTO	fat mass and obesity associated is encoded by the FTO gene
GHRL	Ghrelin And Obestatin Prepropeptide is encoded by the GHRL gene
GIT	gastrointestinal tract
GLUT2	Glucose transporter 2 is encoded by the GLUT2 gene
GM-CSF	Granulocyte-macrophage colony-stimulating factor
GO	Gene Ontology
GPX1	Glutathione peroxidase 1 is encoded by the GPX1 gene
HbA1c	glycated hemoglobin
HDAC1	histone deacetylase 1 is encoded by the HDAC1 gene
HDL	high-density lipoproteins
HDL-C	high-density lipoproteins-cholesterol
ICAM1	intercellular adhesion molecule is encoded by the ICAM1 gene
IFNγ	interferon gamma
IL-1, 2, 5, 6, 7, 8, 10, 13, 15, 17	Interleukin 1, 2, 5, 6, 7, 8, 10, 13, 15, 17 encoded by the same genes
IL-1Ra	interleukin-1 receptor antagonist is encoded by the IL-1Ra gene
IL7R	Interleukin-7 receptor is encoded by the IL7R gene
iNOS	Nitric oxide synthase
KD	Ketogenic diets
KEGG	Kyoto Encyclopedia of Genes and Genomes
Ki67	MKI67 (Marker Of Proliferation Ki-67)
LC3-II/I	Microtubule-associated proteins 1A/1B light chain 3B
LCAT	lecithin-cholesterol acyltransferase is encoded by the LCAT gene
LDL	low-density lipoproteins
LDL-C	low-density lipoprotein-cholesterol
LDLR	low density lipoprotein receptor is encoded by the LDLR gene
LEPR	Leptin Receptor is encoded by the LEPR gene
LGI	low glycemic index
LIPC	Lipase C is encoded by the LIPC gene
LOX-1	lectin-type oxidized LDL receptor 1 is encoded by the OLR1 (oxidized low density lipoprotein receptor 1) gene
LPL	lipoprotein lipase is encoded by the LPL gene
LT-α	Lymphotoxin-alpha (LTA) is encoded by the LTA gene
LXRα	Liver X receptor alpha is encoded by the NR1H3 gene (nuclear receptor subfamily 1, group H, member 3).
MC4R	Melanocortin 4 receptor is encoded by the MC4R gene
MCF-7 cell	michigan cancer foundation-7 (breast cancer cell line)
MCP-1	Monocyte Chemoattractant Protein 1
M-CSF	macrophage colony stimulation factor
MD	Mediterranean Diet
MDA	malondialdehyde
MDA-MB-231 cell line	epithelial, human breast cancer cell line
microRNAs	small single-stranded non-coding RNA molecule
MMP12	Matrix metalloproteinase-12 is encoded by the MMP12 gene
MMP-2	matrix metallopeptidase 2 is encoded by the MMP2 gene
MRI	magnetic resonance imaging
mRNA	messenger ribonucleic acid
MTHFR	Methylenetetrahydrofolatereductase is encoded by the MTHFR gene
mTOR	mammalian target of rapamycin
MTTP	microsomal triglyceride transfer protein is encoded by the MTTP gene
NAD	nicotinamide adenine dinucleotide
NF-κB	nuclear factor kappa-light-chain-enhancer of activated B cells
ox-LDL	oxidized low-density lipoprotein
PCSK9	Proprotein convertase subtilisin/kexin type 9 is encoded by the PCSK9 gene
PECAM	Platelet endothelial cell adhesion molecule is encoded by the PECAM gene
PI3K/AKT/mTOR	PI3K/AKT/mTOR pathway
PI3Ks	Phosphoinositide 3-kinases
PON1	increased paraoxonase is encoded by the PON1 gene
PPARγ2	Peroxisome proliferator-activated receptor γ2 is encoded by the PPARγ2 gene
RNA	Ribonucleic acid
SCD1	stearoyl-Coenzyme A desaturase 1 is encoded by the SCD1 gene
SCFAs	Short-chain fatty acids
SIRT1	NAD-dependent deacetylase sirtuin-1 is encoded by the SIRT1 gene
SMC	smooth muscle cells
SNP	single nucleotide polymorphism
SR-AII	Macrophage scavenger receptor 1 (MSR1) is encoded by the MSR1 gene
SREBP-1	Sterol regulatory element-binding transcription factor 1 is encoded by the SREBP-1 gene
SOD	Superoxide dismutase is encoded by the SOD gene
TAS1R2	Taste receptor type 1 member 2 is encoded by the TAS1R2 gene
TAS2R38	Taste receptor 2 member 38 is encoded by the TAS2R38 gene
TG	triglycerides
TGFβ	Transforming growth factor beta
TH2 cells	T helper 2 cells
THP-1	monocytic cell line
TIGIT	T cell immunoreceptor with Ig and ITIM domains is encoded by the TIGIT gene
TLR4	toll like receptor 4 is encoded by the TLR4 gene
TMA	trimethylamine
TMAO	trimethylamine oxide
TNF-receptor	Tumor necrosis factor receptor is encoded by the TNFRSF1A gene
TNF-α	tumor necrosis factor alpha is encoded by the TNF gene
TSPYL2	Testis-specific Y-encoded-like protein 2 is encoded by the TSPYL2 gene
USA	The United States of America
VACM1	vascular cell adhesion molecule 1 is encoded by the VACM1 gene
VEGF	vascular endothelial growth factor is encoded by the VEGF gene
VSMCs	vascular smooth muscle cells
WOS	Web of Science
